# Probing biological activity through structural modelling of ligand-receptor interactions of 2,4-disubstituted thiazole retinoids

**DOI:** 10.1016/j.bmc.2018.02.002

**Published:** 2018-05-01

**Authors:** Hesham Haffez, David R. Chisholm, Natalie J. Tatum, Roy Valentine, Christopher Redfern, Ehmke Pohl, Andrew Whiting, Stefan Przyborski

**Affiliations:** a Department of Biochemistry and Molecular Biology, Pharmacy College, Helwan University, Cairo, Egypt; b Department of Chemistry, Centre for Sustainable Chemical Processes, Durham University, South Road, Durham DH1 3LE, UK; c Northern Institute for Cancer Research, Medical School, Newcastle University, Newcastle upon Tyne NE2 4HH, UK; d High Force Research Limited, Bowburn North Industrial Estate, Bowburn, Durham DH6 5PF, UK; e Department of Biosciences, Durham University, South Road, Durham DH1 3LE, UK

**Keywords:** ATRA, all-*trans* retinoic acid, AF, activation function, ESI, electronic supplementary information, GZ, compound series code, H12, helix 12, LBD, ligand binding domain, RAR, retinoic acid receptor, RARE, retinoic acid response element, RXR, retinoid X receptor, TTN, 1,1,4,4-tetramethyl-1,2,3,4-tetrahydronaphthalene hydrophobic region

## Abstract

Retinoids, such as all-*trans*-retinoic acid (ATRA), regulate cellular differentiation and signalling pathways in chordates by binding to nuclear retinoic acid receptors (RARα/β/γ). Polar interactions between receptor and ligand are important for binding and facilitating the non-polar interactions and conformational changes necessary for RAR-mediated transcriptional regulation. The constraints on activity and RAR-type specificity with respect to the structural link between the polar and non-polar functions of synthetic retinoids are poorly understood. To address this, predictions from *in silico* ligand-RAR docking calculations and molecular dynamics simulations for a small library of stable, synthetic retinoids (designated GZ series) containing a central thiazole linker structure and different hydrophobic region substituents, were tested using a ligand binding assay and a range of cellular biological assays. The docking analysis showed that these thiazole-containing retinoids were well suited to the binding pocket of RARα, particularly *via* a favorable hydrogen bonding interaction between the thiazole and Ser232 of RARα. A bulky hydrophobic region (*i.e.,* present in compounds GZ23 and GZ25**)** was important for interaction with the RAR binding pockets. Ligand binding assays generally reflected the findings from *in silico* docking, and showed that GZ25 was a particularly strongly binding ligand for RARα/β. GZ25 also exhibited higher activity as an inducer of neuronal differentiation than ATRA and other GZ derivatives. These data demonstrate that GZ25 is a stable synthetic retinoid with improved activity which efficiently regulates neuronal differentiation and help to define the key structural requirements for retinoid activity enabling the design and development of the next generation of more active, selective synthetic retinoids as potential therapeutic regulators of neurogenesis.

## Graphical abstract

**Figure:**
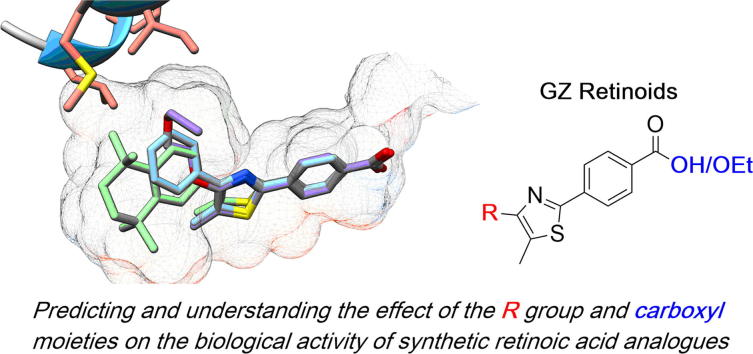

## 1 Introduction

**Fig. 1:**
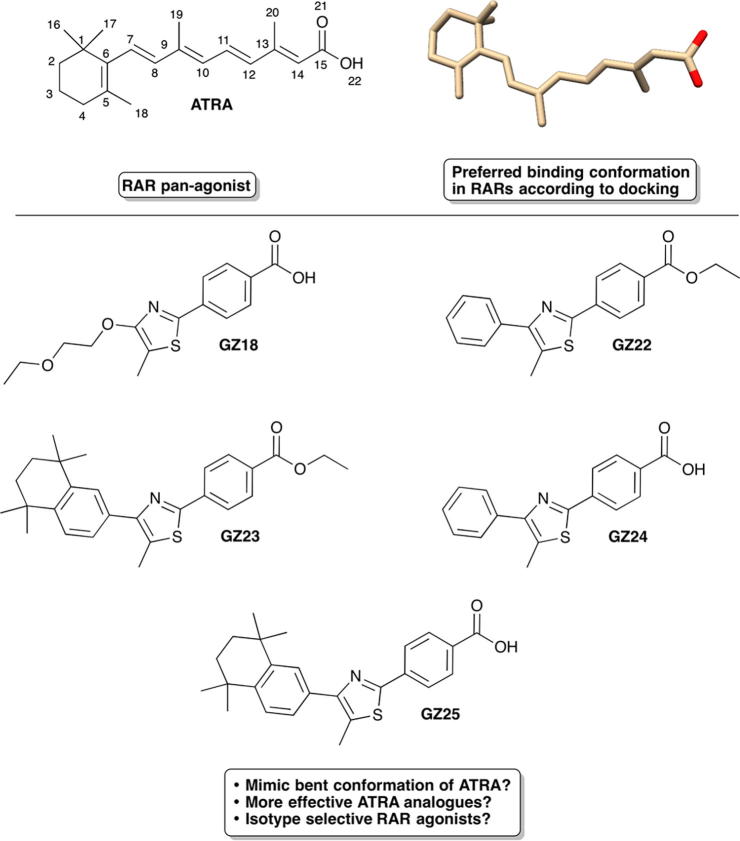
Molecular structures of all-*trans*-retinoic acid (ATRA) and the synthetic thiazole retinoids GZ18, GZ22, GZ23, GZ24 and GZ25, and preferred binding conformation of ATRA according to previous molecular docking studies.^23^

Retinoids are signaling molecules functionally related to all-*trans* retinoic acid (ATRA), a metabolite of Vitamin A (Fig. 1).^1^, ^2^ These small lipophilic molecules mediate cellular proliferation, differentiation and homeostasis in chordates^3^ by acting as ligands for members of a family of nuclear receptors referred to as retinoic acid receptors (RARs) and retinoid X receptors (RXRs). Given the range of biological processes regulated by retinoids, there is huge potential for synthetic retinoids as therapeutics. However, this potential has yet to be realized, mainly because of a lack of detailed understanding of RAR signaling mechanisms in biological processes and the design criteria for targeting synthetic retinoids to specific responses. Furthermore, although retinoic acid is used to treat a variety of skin conditions, acute promyelocytic leukemia, neuroblastoma and other cancers and metabolic diseases,^4^, ^5^, ^6^, ^7^ it is highly susceptible to photodegradation and readily isomerizes to a mixture of 9-*cis*-retinoic acid, 13-*cis*-retinoic acid and other isomers, as well as undergoing decomposition. The development of stable ATRA analogues is, therefore, of substantial importance for improving the potential of RAR signaling pathways for drug development.^8^, ^9^

Three closely related isotypes of each of the RARs (and RXRs) are known, and are designated RARα, RARβ, and RARγ, respectively.^1^ They all share the same overall structure, with six domains, specified A to F.^1^, ^10^ The A and B domains comprise a ligand independent activation function (AF-1), responsible for recruiting coactivators necessary for gene regulation.^11^ The DNA-binding C domain^12^, ^13^ is linked, *via* a hinge domain D, to the ligand-binding domain (LBD) E. The sequences of RAR isotypes are highly conserved, but subtle differences in the LBD give rise to distinct ligand binding pocket architectures, allowing for the design of isotype-specific retinoids.^14^, ^15^ In addition, alternative splicing of primary RAR transcripts generates, for each RAR isotype, subtypes with different A and B domains linked to the RARα-, RARβ- and RARγ-specific core C, D, E and F domains.

The activation mechanism of the RARs (and RXRs) by retinoids has been studied, in detail, by determination of a number of crystal structures of the LBDs. Upon retinoid binding to the LBD, a conformational change occurs allowing heterodimerisation to an RXR partner, and subsequent binding to DNA sequences known as retinoic acid response elements (RAREs).^4^, ^10^, ^16^, ^17^, ^18^ A ‘mouse-trap’ process is thought to occur during retinoid binding to the RARs, in which the retinoid enters the pocket and associates with a cluster of polar residues at the end, facilitating a conformational change which brings the C-terminal helix 12 (H12) to enclose the ligand inside the pocket.^10^ The interaction between H12 and the retinoid ligand is particularly important, since the *holo* positioning of H12 forms a binding surface (AF-2) that binds coactivators.^10^, ^19^, ^20^ Effective stabilization of H12, therefore, leads to the promotion of gene transcription, and indeed, optimization of this interaction has become an important aim for the design of synthetic retinoids.^21^

The molecular structure of natural and synthetic retinoids can be described in terms of three distinct regions: a polar region (typically a carboxyl moiety) required for association with the polar cluster at the base of the pocket, a short linker that increases the length of the retinoid and can incorporate additional functionality for isotype-specific binding, and a hydrophobic region for interacting with H12 and the strongly hydrophobic opening to the binding pocket.^21^, ^22^ In recent molecular docking studies and receptor-binding assays, we showed that ATRA is able to bind to each of the RARs via a number of conformations with respect to the s-*cis* and s*-trans* orientations around formal single bond connections between double bonds. Indeed, a bent conformation with a single s-*cis* bond rotation about the C7–C8 alkene was the preferred conformation over the more linear, all-*trans* conformation frequently thought to be the ‘active’ binding conformation; a conclusion clearly supported by employing a novel clustering conformer analysis approach which is ideal for examining conformationally-flexible ligands bound to protein receptors.^23^, ^24^

The length and shape of synthetic retinoid molecules significantly affects biological activity.^9^ Polar interactions with the carboxyl moiety are important for anchoring the retinoid within the pocket, but the contributions to biological activity and RAR-type selectivity of more-subtle substituent and conformational properties of ligands is not well understood. A strategy for the design of RARα-selective agonists has been the incorporation of a hydrogen bond accepting moiety in the centre of the molecule to form a hydrogen bond with the RARα-specific residue, Ser232.^14^, ^20^, ^21^, ^25^ Furthermore, the greater space available in the binding pocket of RARβ has implications for the design of synthetic compounds with greater specificity for this RAR type.

To probe the relationships between structure and function of synthetic retinoid-like molecules we have used compounds built around a central 2,4-disubstituted thiazole linker; this thiazole sub-structure imposes a slight twist in the overall shape of the molecule which may mimic the preferred binding conformation of ATRA^23^ and may confer selectivity for RARα via interactions with Ser232.^26^, ^27^, ^28^ Members of this synthetic retinoid series (designated GZ, the synthesis of which is reported previously^29^) have either polyalkoxyalkyl (GZ18), phenyl (GZ24 and the ester GZ22) or 1,1,4,4-tetramethyl-1,2,3,4-tetrahydronaphthyl (GZ25 and the ester GZ23) hydrophobic regions (Fig. 1). The angle of the 1,1,4,4-tetramethyl-1,2,3,4-tetrahydronaphthalene (TTN) hydrophobic region with respect to the rest of the retinoid structure could favour orientation towards the larger cavity specific to the ligand binding pocket of RARβ;^30^ indeed, GZ18 has been reported previously as an RARβ2-specific retinoid.^31^ Furthermore, the increased rigidity compared to ATRA, preorganization and lipophilicity of these types of compounds (particularly GZ23 and GZ25) could have entropic and enthalpic benefits for RAR binding,^21^, ^32^ properties that would make the GZ series an ideal platform for the design of RAR-specific ligands as novel retinoid-based drugs.

The aim of this study was to test these predictions for the binding activity and specificity of synthetic retinoids based around the use of a central thiazole linking the carboxyl and non-polar ends. To do this, a molecular docking approach^33^ was used to determine the theoretical binding modes of these compounds, and their predicted activity was tested *via* a ligand-binding assay dependent upon both ligand binding and coactivator recruitment, and compared with biological assays of different types.

## 2 Results and discussion

### 2.1 Molecular modeling, docking and binding analysis

**Fig. 2:**
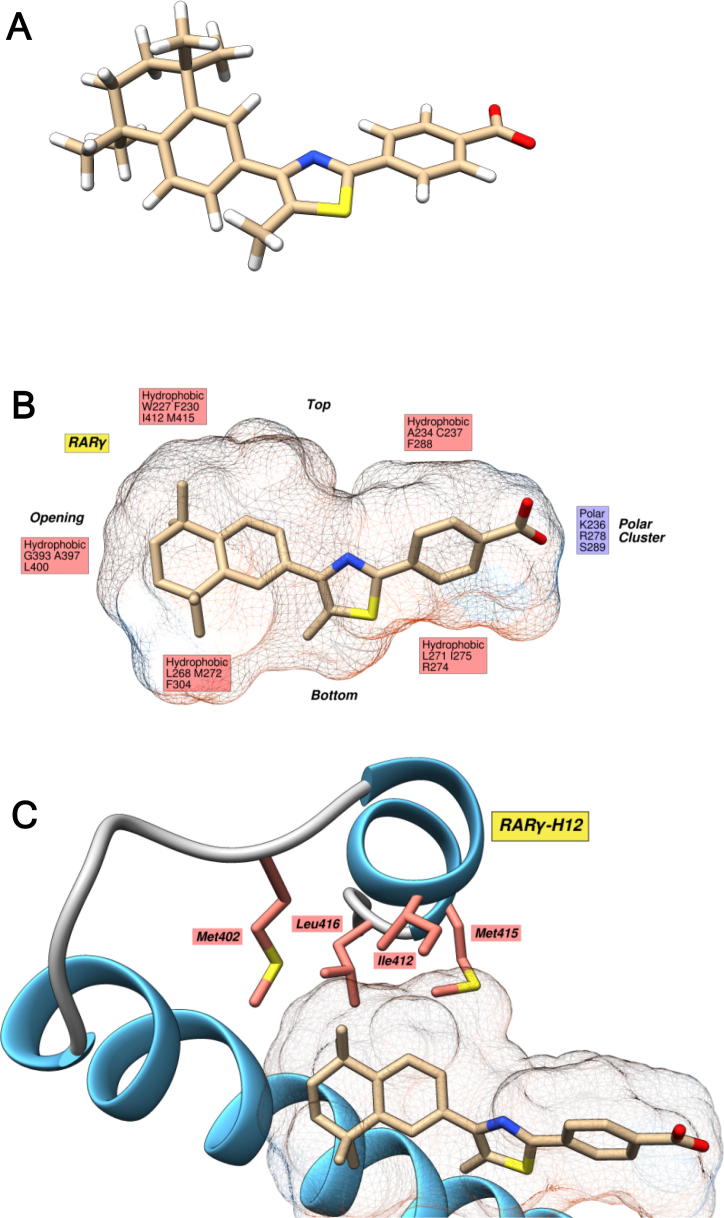
Modelling the interaction between GZ25 and RARγ. A, Lowest energy conformation of GZ25 according to *in vacuo* AM1 conformation distribution calculations. B, Ligand interaction diagram of the highest scoring docking solution of GZ25 in the RARγ (PDB: 2LBD) binding pocket. The hydrophobicity surface of the binding pocket is shown, and highlights the pocket’s predominantly hydrophobic nature. A cluster of polar residues (Lys236, Arg278 and Ser289, in RARγ) at the end of the pocket anchor the retinoid via the carboxylate. Hydrogen atoms are omitted from this and all following images for clarity. C, Key residues involved in the interaction between H12 (RARγ) and the retinoid hydrophobic region. Stabilization of this helix is key to providing a suitable platform for the transcription of RAREs *via* AF-2.

To predict the suitability of GZ18, GZ24 and GZ25, and the corresponding esters GZ22 and GZ23, for binding to the RARs, the molecular docking method we described previously was used.^23^ This involved an initial, semi-empirical calculation of the conformational distribution of each compound to indicate the low energy conformations that the ligand can adopt, and to provide starting points for the molecular docking calculations used to predict the possible binding modes when complexed with the RAR LBDs. A widely-employed docking strategy involves using a number of input conformations for each ligand, rather than a single, low energy conformation and allowing maximum ligand flexibility in each independent docking simulation. This approach enables a more complete assessment of the potential conformational space that can be occupied by the ligand when bound to a target, and improves the probability of finding a global energy minimum.^34^, ^35^, ^36^ Accordingly, the molecular structure of each GZ retinoid was generated and used as the basis for a conformational distribution calculation using the AM1 force field.^37^, ^38^ For each low energy conformation generated, the central thiazole linker was found to be coplanar with the benzoate polar region. The phenyl and TTN hydrophobic region of the aryl-substituted retinoids (GZ22, GZ23, GZ24, GZ25) exhibited a 40° dihedral angle with respect to the rest of the molecule (Fig. 2A) but the glycol hydrophobic region of GZ18 exhibited a high degree of conformational flexibility (all starting conformations for the docking, and X-ray crystal structures of GZ18, GZ22 and GZ23 are shown in the ESI).

Each starting conformation was then docked using GOLD^39^ into the receptor binding sites utilising structures derived from the highest resolution crystal structures of RARα,^20^ RARβ^30^ and RARγ^10^ available in the PDB. From each input conformation of each ligand, three to ten docking solutions were returned and then examined individually before clustering (see ESI).

#### 2.1.1 Key interactions

The RAR binding pocket is highly hydrophobic and the interactions formed between the protein and the retinoid ligand are, therefore, mainly hydrophobic in nature (Fig. 2B).^15^, ^30^ Conversely, a cluster of polar residues at the end of the pocket (Lys236, Arg278 and Ser289 in the case of RARγ) perform an important role in guiding the retinoid into the pocket *via* the carboxylate.^10^ This, in turn, forms a salt bridge and hydrogen bonding interactions with this polar cluster that are important for high binding affinity.^23^ The most important ligand-binding motif in regards to transcriptional activation activity is the interaction with H12 because ligand-dependent stabilisation of this helix in the *holo* position is required for coactivator recruitment.^11^, ^20^, ^40^ H12 is also mainly hydrophobic (Fig. 2C) and is key in the interaction (principally *via* interactions with Met402, Ile412, Met415 and Leu416 in RARγ) with the hydrophobic region of the retinoid.

#### 2.1.2 RARα

**Fig. 3:**
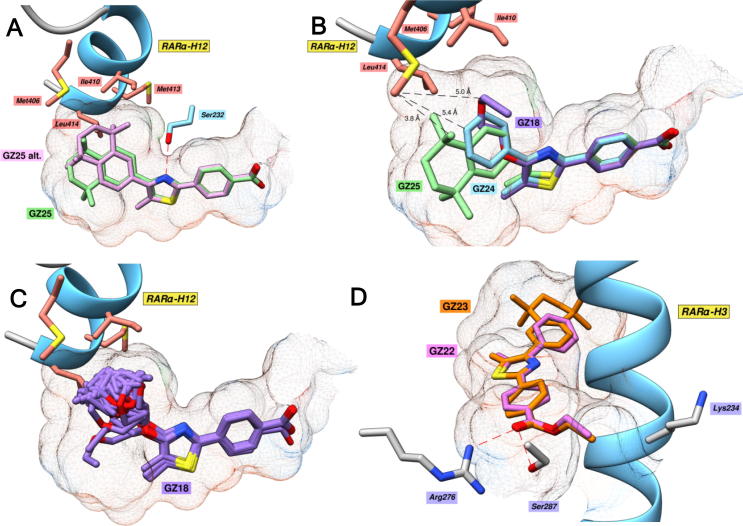
Modeling the interactions between GZ series retinoids and RARα. A, Highest scoring docking pose (green) of GZ25 docked into RARα (PDB: 3KMR). A single docking solution (pink) also indicated an alternative positioning of the hydrophobic region in which the TTN ring system is oriented upwards directly towards H12. B, Comparison of the highest scoring docking poses of GZ18, GZ24 and GZ25 in RARα. C, Structural overlay of each docking solution (50 solutions) of GZ18 in RARα. D, Highest scoring docking solutions of GZ22 and GZ23 in RARα.

The docking analysis clearly showed a hydrogen bonding interaction (2.7–3.0 Å) between the thiazole nitrogen and Ser232 (Fig. 3A). This interaction forces the thiazole ring to be oriented with the nitrogen pointing upwards in all docking solutions; a positioning which facilitates interactions between the hydrophobic region and H12. In addition, the positioning of the benzoate polar region was similar for all solutions for each GZ retinoid. In the case of GZ25, each of the calculated docking poses (13 solutions were returned) were similar (Fig. 3A), apart from a single pose which exhibited an alternative positioning of the hydrophobic region which was orientated directly towards H12. This enhances interactions with Met406 on H12 (3.5 Å compared to 4.2 Å) but causes a steric clash with Ile410 and sacrifices contacts with the bottom of the pocket, particularly with Leu305 (4.3 Å compared to 3.2 Å). Overall, our results show that GZ25 is well-suited to the binding pocket of RARα.

In contrast, the hydrophobic regions of GZ18, GZ24 and GZ25 display marked differences in the extent of interactions with H12. The much larger TTN hydrophobic region of GZ25 formed more-extensive hydrophobic interactions (Fig. 3B) than GZ18 and GZ24, particularly with Met406, which requires stabilization to maintain the closed, *holo*, conformation of the loop region between H11 and H12.^10^, ^19^ The hydrophobic region of GZ18 adopts a number of different conformations because of the high flexibility of this region (Fig. 3C). This flexibility may impose a significant entropic penalty upon binding to this relatively fixed binding pocket, particularly in comparison to the more rigid, preorganized structures of GZ24/GZ25. Moreover, while the phenyl group of GZ24 was unable to associate with the majority of the hydrophobic residues at the opening of the pocket like the TTN of GZ25, the docking simulation did indicate improved hydrophobic interactions with the binding pocket of RARα compared to GZ18. Accordingly, we would expect GZ25 to exhibit the highest binding affinity for RARα, followed by GZ24, and then GZ18.

The esters, GZ22 and GZ23 exhibited very similar positions to their carboxylic acid counterparts according to the docking calculations. The hydrophobic regions formed similar contacts with H12 and the highly nonpolar opening to the pocket, and the thiazole linker is well positioned for hydrogen bonding to Ser232. However, the ethyl ester moiety occupies a slightly different positioning compared to the carboxylate of the other retinoids, in which the ethyl group is pushed into a small cavity formed between Ser287 and Lys234 (Fig. 3D). This positioning is not unfavorable, *per se*, however, the result is to slightly increase the distance to the guanidine moiety of the key polar residue Arg276 (3.1 Å for GZ23 versus 2.8 Å for GZ25), and inhibits the ability the form a hydrogen bonding interaction with the Ser287 sidechain hydroxyl. Therefore, based on these findings, the esters GZ22 and GZ23, would be expected to exhibit reduced binding affinity for RARα compared to the corresponding acids, GZ24 and GZ25.

#### 2.1.3 RARβ

**Fig. 4:**
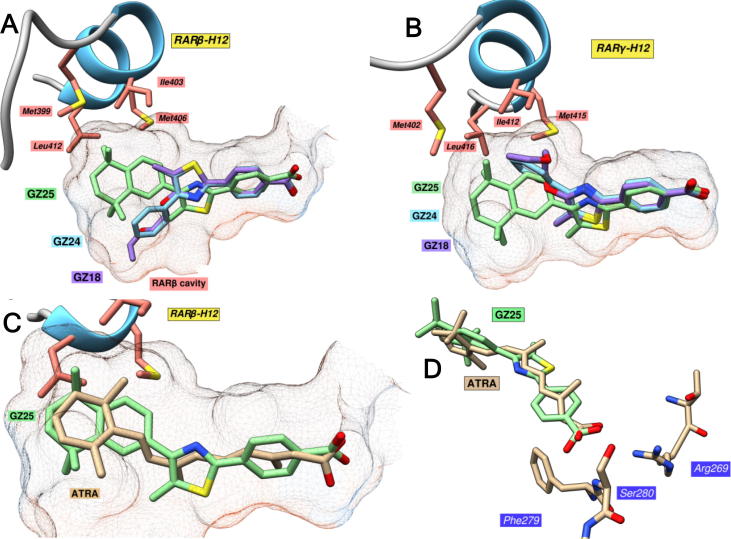
Interactions between the GZ retinoids and RARβ and RARγ. A, Highest scoring docking solutions of GZ18, GZ24 and GZ25 in RARβ. B, Highest scoring docking solutions of GZ18, GZ24 and GZ25 in RARγ. C, Comparison between the highest scoring docking solutions of GZ25 and ATRA in RARβ.^23^ D, Comparison of the carboxylate positioning of the highest scoring docking solutions of GZ25 and ATRA in RARβ.

The ligand binding pocket of RARβ is significantly larger than in RARα or RARγ (503.5 Å^3^ versus 429.4 Å^3^ for RARγ) because of the additional cavity around the opening of the pocket (Fig. 4A).^15^, ^30^ RARβ-specific ligands have been designed by incorporating larger hydrophobic regions that can fill this cavity.^21^, ^30^ When the carboxylic acids, GZ18, GZ24 and GZ25, were docked into RARβ, a broader range of docking poses was obtained in comparison to in RARα. While the positioning of the polar region was fairly similar in each solution, in RARβ, the thiazole ring was oriented with the nitrogen pointing upwards or downwards. Consequently, this orientates the hydrophobic region either upwards towards H12, or downwards towards the RARβ-specific cavity. The preference of each ligand for these two orientations was assessed by examining each docking solution individually (see ESI). GZ18 favored orientation of the hydrophobic region downwards, presumably because this allows a fully extended conformation of the glycol structure. This orientation may, however, sacrifice interactions with H12, thus suggesting that GZ18 may be a RARβ-specific retinoid,^31^ but one that is less capable of stabilizing H12 for coactivator binding.^19^, ^41^

GZ24 also docked exclusively with the hydrophobic region pointing downwards towards this specific cavity, though the shorter phenyl hydrophobic region formed fewer and weaker contacts with this area of the binding pocket (distance to Ile380: GZ18 = 3.3 Å, GZ24 = 5.2 Å, distance to Val302: GZ18 = 3.3 Å, GZ24 = 4.6 Å), suggesting that GZ24 will have a lower affinity for RARβ than GZ18.^30^ In contrast, GZ25 exhibited an almost complete preference for the hydrophobic region to be pointed upwards towards H12, presumably since the much larger size of this structure mediates particularly strong contacts with this helix. Given this, one can predict that GZ25 should exhibit strong, but not necessarily specific binding to RARβ. Also, as in RARα, the esters, GZ22 and GZ23, exhibited similar docking poses to the corresponding carboxylic acids, GZ24 and GZ25, though the ester group elicits weaker polar interactions with the polar cluster at the end of the pocket. Reduced binding affinity for the esters was, therefore, predicted based on these docking results.

#### 2.1.4 RARγ

As with RARα, each GZ retinoid exhibited docking poses in the binding pocket of RARγ where the thiazole ring was oriented with the nitrogen atom pointing upwards, presumably because the opposite orientation would elicit unfavorable clashes between the hydrophobic region and the bottom of the pocket. Furthermore, GZ18, GZ24 and GZ25 each adopted similar poses to those observed in RARα (Fig. 4B); however, GZ25 was positioned slightly lower in the pocket to avoid clashes with Ala234 and Leu271 around the middle of the top surface of the binding pocket. The positioning was particularly favorable for GZ25, and the hydrophobic region filled the wider opening of the binding pocket well, forming more extensive hydrophobic contacts with Met402 (3.4 Å) and Leu416 (3.2 Å) on H12 when compared to GZ18 (5.6 Å and 4.8 Å, respectively) and GZ24 (5.3 Å and 4.7 Å, respectively). However, this lower positioning resulted in clashes between the thiazole sulfur and the RARγ-specific residue Met272, and between the thiazole methyl and the nearby Ile275. This observation may suggest that the central thiazole linker is less suited to RARγ than RARα/β. Similarly to RARα, the GZ22 and GZ23 esters adopted similar poses in the RARγ LBD to their carboxylic acid counterparts, but the ester groups formed poorer contacts with the polar cluster at the end of the pocket. This was particularly apparent with GZ23, whose ethyl group was forced away from the polar cluster and directly towards Cys237 on H3, resulting in a strong clash. Therefore, GZ22 and GZ23 may well exhibit reduced binding affinity for RARγ compared to the corresponding carboxylic acids.

#### 2.1.5 Binding of GZ25 compared to ATRA

There was a high degree of similarity between the highest scoring docking solutions for GZ25 and ATRA (Fig. 4C).^23^ The cyclohexenyl hydrophobic region of ATRA was twisted from the continuity of the polyene chain at almost the same angle as between the planes of the GZ25 hydrophobic region and the linker thiazole ring. However, the increased size of the hydrophobic, linker and polar regions of GZ25 broadly means that GZ25 fills the pockets of the RARs more completely than ATRA and, particularly in the case of the smaller RARα/γ, elicits more extensive contacts with the bottom of the pocket. The carboxylates of GZ25 and ATRA show some differences; while the ATRA carboxylate is oriented at an angle that benefits both hydrogen bonding with Ser280 and salt bridge formation with Arg269, that of GZ25 is positioned slightly further from Ser280 (3.4 Å versus 2.9 Å) and at a less favorable angle for hydrogen bonding (Fig. 4D). However, this is compensated for by the formation of a hydrogen bonding interaction with the main chain amide of Phe279-Ser280.

#### 2.1.6 Comparison of docking methods with molecular dynamics simulation

**Fig. 5:**
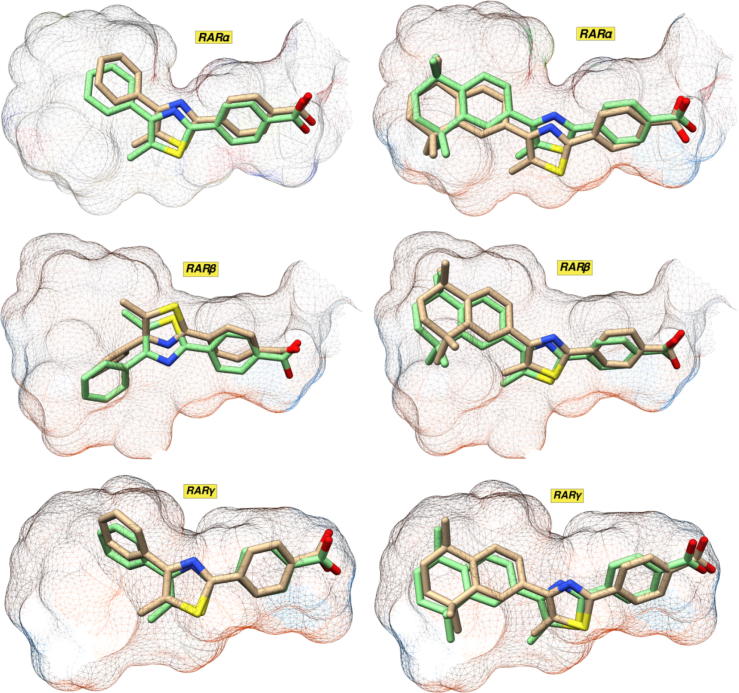
Comparison between the docking binding poses (green) and the MD binding poses (tan) for GZ24 (left) and GZ25 (right) in each of the RARs.

In order to corroborate the results from the docking studies, we conducted molecular dynamics (MD) simulations involving the full RAR LBDs and the GZ retinoids, using GROMACS.^42^, ^43^ Each of the docked structures were used as starting points for MD simulations computed over 10 ns. These simulations showed that, over this timescale, only very minor protein movements were observed, indicating that the RAR PDB crystal structures were good starting points for molecular docking calculations. A comparison (Fig. 5) between the docked and MD binding poses for GZ24 and GZ25 (other compounds shown in the ESI) in each of the RARs showed that the ligand binding poses from MD generally matched those from the docking very well, with an exception being the docking versus MD results for GZ22 in RARβ and RARγ. In this case, the MD pose ignored interactions between the carboxy moiety of the ligand and the polar cluster, and instead, moved the hydrophobic region closer to the hydrophobic opening of the pockets. This alternative binding pose may be due to an overestimation of the potential stabilisation gained from interacting with the hydrophobic opening in favour of the likely stronger (in reality) polar interactions at the opposite end of the pocket. Therefore, given the lipophilicity of the ligands and the well-established difficulties of methods such as MM/PBSA to account for entropic effects, we used the MD simulations to validate the docking predictions, rather than as a means to predict the binding energies/affinities of the GZ retinoids.^44^ Importantly, however, the MD versus docking comparison showed that our molecular docking protocol provided informative and meaningful binding poses that were directly comparable with those calculated at a higher level of theory, with the major advantage being that docking can be completed in a fraction of the computation time.

### 2.2 Ligand-receptor assay

The main conclusions and predictions from the computational binding studies are that: 1) GZ25 would exhibit greater binding affinity for RARα/β/γ than each of the other acids, GZ18 and GZ24, and the esters GZ22 and GZ23; 2) each of the GZ retinoids may exhibit specificity for RARα due to a hydrogen bonding interaction with Ser232; 3) the TTN hydrophobic region of both GZ23/GZ25 forms much more extensive contacts with H12 in each of the RARs compared to the smaller hydrophobic regions of the other GZ retinoids, which may well translate to improved transcriptional activity; 4) the ethyl ester group of GZ22 and GZ23 may reduce the strength of interaction with the polar cluster at the end of the pocket, thus reducing binding affinity; 5) the lower positioning of the thiazole linker in RARγ may cause clashes with the bottom of the binding pocket that could translate to reduced binding affinity; 6) GZ25 and ATRA show major similarities in overall binding conformation and positioning, together with some differences in polar interactions.

**Fig. 6:**
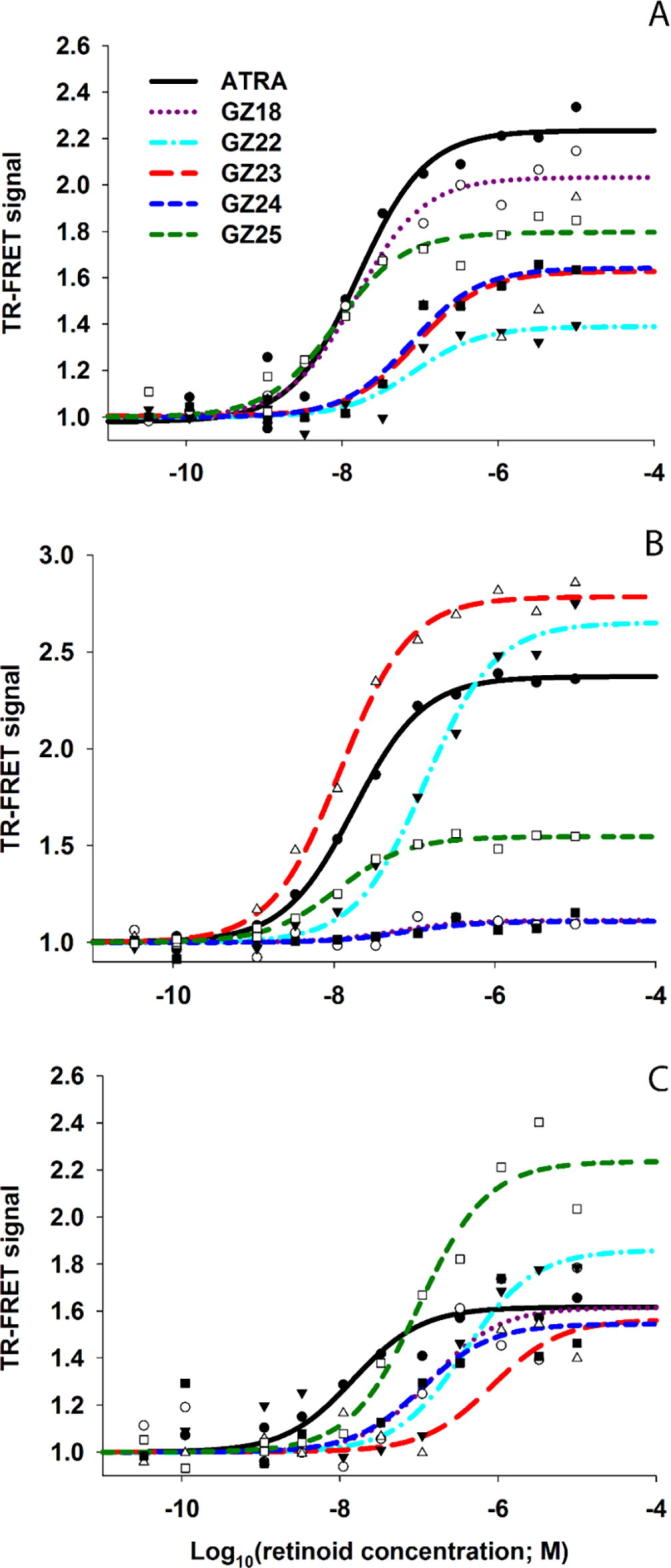
Testing the ability of GZ series retinoids to bind RARα (A), RARβ (B) and RARγ (C) and recruit coactivator.

These predictions were tested through a receptor-binding assay for each RAR isotype (Fig. 6), where the assay signal is dependent on two parameters: the binding affinity of ligand to the RAR LBD, and the affinity of the coactivator for the ligand-LBD complex. For this assay, binding curves and variation in level of the upper asymptote can reflect differences in co-activator affinity for the ligand-LBD complex; variation in affinity of ligand for LBD shifts the midpoint (EC_50_) of the binding curve, whereas changes in the level of the upper asymptote may result from changes in coactivator affinity for the ligand-LBD complex alone, or in combination with changes in affinity for ligand with LBD.^23^

Binding activity demonstrated variation by RAR type (Table 1, Fig. 6). For RARα the retinoids fell into two groups: (1) ATRA, GZ18 and GZ25 with good binding affinity (low EC_50_) and high curve asymptotes (Fig. 6), (2) GZ22, GZ23, GZ24 with poor binding (high EC_50_) and lower asymptotes. There was greater variability with respect to RARβ, with low EC_50_ and high asymptotes for GZ23 and ATRA, high EC_50_ and high asymptote for GZ22, and high EC_50_ with very low asymptotes for GZ24 and GZ18. For RARγ, asymptotes ranged from intermediate (GZ23/GZ24) to high (GZ25), with a low EC_50_ values only for ATRA, intermediate values for GZ25, GZ18 and GZ24, but high values for GZ23 and GZ22.

**Table 1:** Estimated EC_50_ values (95% confidence ranges in parentheses) for ATRA, GZ18, GZ22, GZ23, GZ24 and GZ25 with RARα, RARβ and RARγ.

Retinoid	EC_50_ [nM] (95% confidence range)		
	RARα	RARβ	RARγ
ATRA	16.1 (8.8–29.4)	17.6 (15.2–20.3)	14.7 (5.4–39.8)
GZ18	15.1 (9.5–24)	56.7 (2–1591)	141.3 (25.6–778.1)
GZ22	82 (25.3–265.7)	136.3 (99.4–186.8)	383 (96.7–1517)
GZ23	88.9 (15.5–511.7)	12.1 (9.4–15.5)	847.2 (99.5–7211)
GZ24	77.5 (41.6–144.2)	77.8 (4.5–1338)	109.2 (13.8–863.7)
GZ25	8.2 (3.9–17.3)	11 (7.7–15.7)	98.9 (51.6–189.6)

Thus, the carboxylic acids, GZ18, GZ24 and GZ25 exhibit high affinity for each of the RARs, but with varying degrees of selectivity. GZ25 was generally more potent than ATRA for RARα/β, but the lower affinity for RARγ may be compensated by the greater coactivator recruitment activity. These results, and the overall lower affinity of GZ24 for the RARs, was in good agreement with the docking predictions. Conversely, GZ23, the ester of GZ25 had a surprising selectivity for RARβ, suggesting that the attenuation of interactions with the polar cluster of the LBD is less detrimental for this RAR than suggested by the docking, and may be compensated by more-extensive contacts of the TTN hydrophobic region with H12 facilitated by the larger cavity within the RARβ LBD.^23^ The weaker binding interactions of the esters, GZ22 and GZ23, were in general agreement with the docking studies, and the reduced interaction of GZ23 for RARγ compared to GZ25 may be due to the predicted clash with Cys237.

The computational studies highlighted a potentially important hydrogen bonding interaction between the thiazole nitrogen of each GZ retinoid and Ser232 in RARα.^14^ While GZ25 did exhibit the strongest binding affinity in RARα, the idea that this interaction could confer a degree of selectivity for this isotype was not supported by the binding assay data, and this interaction may be beneficial but not crucial as a driver of specificity. GZ18 exhibited some RAR selectivity, but not as much as suggested by other studies.^31^ For this retinoid, confidence limits for the EC_50_ estimates for RARβ and RARγ were wide; a consequence of very low binding-curve upper asymptotes (Fig. 6) indicating a poor ability for the ligand-bound LBD to recruit coactivator, as predicted by the docking analyses. Overall, the wide variation between retinoids in the upper asymptotes of the binding-curves demonstrate the importance of ligand-LBD-dependent interactions with co-activators for retinoid activity. However, even with the type of binding assay employed here, it may not be possible to predict the biological activity of different retinoids because retinoid responses in cellular contexts will vary with patterns of RAR expression in combination with different co-activators. Therefore, biological activity should be measured directly.

### 2.3 Biological activity

Testing the biological activity of novel retinoids is essential for asking whether apparent selectivity in ligand binding translates to retinoid-dependent biological activity*.* The ligand binding assays supported previous reports^29^ that GZ25 exhibits strong biological activity but, for other GZ compounds, the evidence of reduced ability to recruit coactivator raised major questions about likely biological activity. RAR-mediated mechanisms as drivers of biological responses are poorly understood because of the confounding effects of varying RAR specificity of different gene promoters, cellular context and the potential for simultaneous and/or sequential interactions of different RARs. Reliance on a single biological assay is, therefore, likely to be misleading and we have used a range of approaches to assess biological activity with respect to broad- (neuronal differentiation phenotype) and finer-scale responses (gene induction) for our GZ series compounds, with focus on GZ25 and GZ18 in comparison to ATRA.

**Fig. 7:**
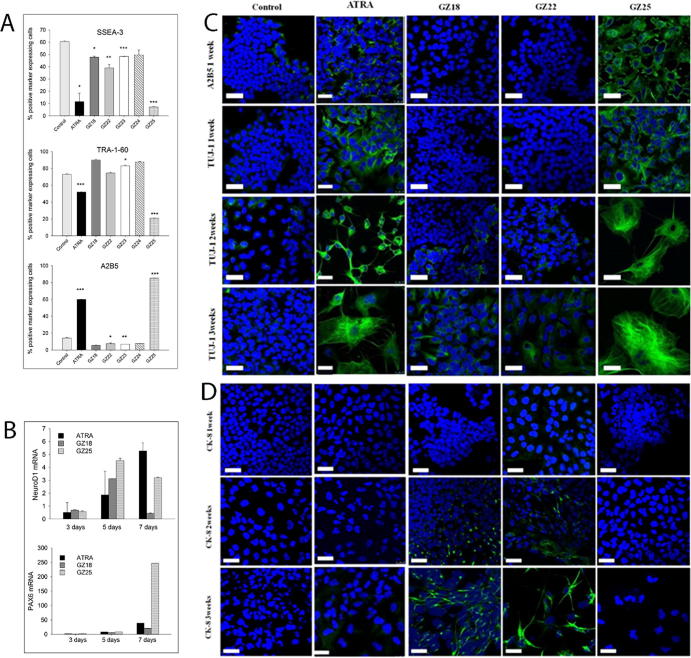
Biological assessment of TERA2.cl.SP12 stem cells treated with GZ series retinoids. A, Flow cytometry (antibody staining; percentage of gated cells positive for the marker) for expression of the cell-surface differentiation markers SSEA-3, TRA-1–60 and A2B5. Data represent mean ± SEM, n = 3; ^*^P < 0.05; ^**^P < 0.01; ^***^P < 0.001, relative to control cells. B, Real-time PCR assay for the neuronal markers NeuroD1 (top graph) and PAX6 (bottom graph) in TERA2.cl.SP12 stem cells treated with 10 μM of ATRA, GZ18 or GZ25 for 3, 5 and 7 days, relative to TERA2.cl.SP12 cells treated with 0.1% DMSO vehicle for 7 days; all data (mean ± SEM, n = 3) normalized to the internal reference gene (GADPH). C, TERA2.cl.SP12 cells stained for the neuronal markers A2B5 and TUJ-1 after exposure to control vehicle, 10 μM ATRA, GZ18, GZ22 or GZ25 for 1, 2 and 3 weeks; scale bar, 25 μm. D, TERA2.cl.SP12 cells stained for cytokeratin 8 (Ck-8) after exposure to retinoids as in C; scale bar, 25 μm.

First, we examined neuronal differentiation in pluripotent TERA2.cl.SP12 human embryonal carcinoma stem cells in response to retinoid treatment. TERA2.cl.SP12 cells have been used in previous studies^8^, ^29^ to test the ability of synthetic retinoids to induce differentiation along a neuronal or an alternative epithelial pathway at long *in vitro* timescales of up to three weeks. The response to ATRA in these cells can be quantified through changes in the proportion of cells expressing the specific cell surface markers, SSEA-3, TRA-1–60 and A2B5. SSEA-3 and TRA-1–60 are epitopes associated with stem-cell pluripotency^45^, ^46^, ^47^ and typically downregulated as a result of cellular differentiation.^45^ Conversely, A2B5, a neuronal cell-membrane ganglioside antigen, is normally upregulated as a result of neuronal differentiation.^48^ Using flow cytometry, we measured the proportion of cells expressing these differentiation markers after exposure to 10 μM of the different GZ retinoids for 7 days and compared the results to ATRA. GZ25 was the most potent, even compared to ATRA (Fig. 7A), whereas GZ18, GZ23 and GZ24 had very little activity compared to control cultures. Intermediate responses were apparent in cells treated with GZ22. Since the parent carboxylic acid (GZ24) had little activity, the response to GZ22 was a real effect of the ester rather than a result of hydrolysis of the parent ester to the carboxylic acid.

Second, NeuroD1 and PAX6 are retinoid-induced transcription factors involved in regulating neuronal differentiation,^49^, ^50^ and are expressed in neurons derived from TERA2.cl.SP12 stem cells. Therefore, we examined the expression of mRNA for these transcription factors in TERA2.cl.SP12 cells, which showed time-dependent increases after treatment for up to 7 days after exposure to GZ25, GZ18 or ATRA. NeuroD1 was expressed in response to GZ25, G18 or ATRA after 5 days (GZ25 > GZ18 > ATRA), but after 7 days, expression was only maintained in response to ATRA or GZ25 (ATRA > GZ25; Fig. 7B). With respect to the induction of PAX6 expression, GZ25 was substantially more active than either ATRA or GZ18 (GZ25 ≫ ATRA > GZ18).

Third, we carried out assessment of protein expression using immunofluorescent techniques on cultures of TERA2.cl.SP12 cells treated with retinoids. Expression of the neuronal differentiation marker A2B5 was induced after 7 days with GZ25 or ATRA, with greater staining intensity for the GZ25-treated cells (Fig. 7C). Over longer timescales of up to 3 weeks, GZ25 and ATRA were also the most effective at inducing neuronal differentiation, as shown by immunocytochemical staining for the neuronal marker TUJ-1 (neuron-specific class III β-tubulin), with a very low activity of GZ18 or GZ22 only apparent from 2 weeks as small, scattered colonies of TUJ-1-positive cells. In contrast, GZ18 and GZ22 were more effective at inducing the expression of the epithelial phenotype marker cytokeratin 8, which was not detectable in cells treated with ATRA or GZ25 (Fig. 7D).

Fourth, we examined the morphological changes (neurite extension) and relative levels of induction of the neuronal marker seen in the immunofluorescent experiments (Fig. 7C and D). With ATRA and GZ25, they fitted the pattern of activity expected with respect to changes in expression of the stem cell markers and NeuroD1 and PAX6 expression. In respect of epithelial differentiation indicated by the induction of cytokeratin 8, this behavior of GZ18 and GZ22 mirrors that of the 3-(tetramethyl-tetrahydronaphthalenyl-ethynyl)benzoic acid retinoid, EC19.^8^ However, EC19 is known to be relatively selective for RARβ,^23^ whereas GZ18 and GZ22 showed little convincing evidence for RARβ selectivity, either with respect to ligand binding affinity or coactivator recruitment but better selectivity for RARα. Although GZ18 has been described by others^31^ as an RARβ2-isoform-specific ligand, different RARβ isoforms (which vary in the A and B domains) will have the same LBD and such an apparent specificity is likely to be an artefact of the biological RAR-specificity assays employed. At face value and in the context of data for EC19, these results suggest that neural differentiation is dependent on combinatorial activity, either simultaneously or sequentially, by RARα and RARβ, with epithelial differentiation resulting from activation of one receptor type or as a result of a non-specific effect.

**Fig. 8:**
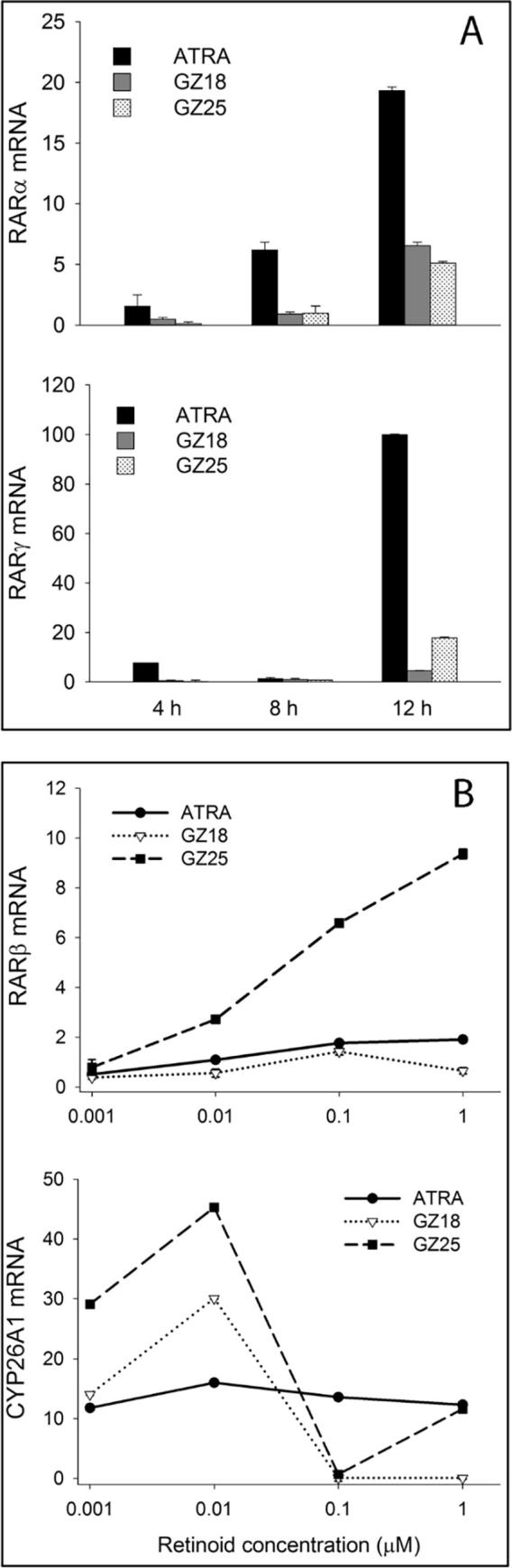
Retinoid-induced gene expression in SH-SY5Y cells treated with ATRA, GZ18 or GZ25. A, expression of RARα and RARγ after 4, 8 or 12 h exposure to 10 μM retinoids. B, expression of RARβ and CYP26A1 in response to retinoid concentrations of 1, 0.1, 0.01 and 0.001 μM for 8 h. Quantification of target mRNA was relative to cells cultured with DMSO vehicle for the relevant time period and normalized to the internal reference gene (ACTB). Data represent mean ± SEM, n = 3.

As an additional assay of biological activity, short-term changes in expression of four genes in SH-SY5Y cells was employed; a neuroblastoma-derived lineage that undergoes neuronal differentiation in response to retinoid treatment. The four genes assayed, RARα, RARβ, RARγ and CYP26A, have RAREs associated with their promoters and may be directly regulated by retinoids.^51^, ^52^ RARα and RARγ were induced in a time-dependent manner over a 12 h period by 10 μM retinoid, with ATRA having the greatest activity and GZ18 and GZ25 giving lower levels of induction (Fig. 8A). Time-courses for RARβ and CYP26A1 induction after 10 μM retinoid treatment were more variable, but GZ18 was less active in comparison to either GZ25 or ATRA. Dose-response experiments over the range 1 nM to 1 μM for these latter two genes (after 8 h treatment), indicated a dose-dependent induction of RARβ with GZ25 having greater activity than either ATRA or GZ18 (Fig. 8B). For CYP26A1, both GZ25 and GZ18 (GZ25 > GZ18) had greater activity than ATRA at doses <1 μM, but less activity than ATRA at higher doses. GZ25 was clearly the most effective retinoid (GZ25 ≫ ATRA > GZ18) at inducing RARβ at all doses (Fig. 8B). RARβ expression is likely to be driven predominantly by RARβ and the relatively low activity of GZ18 contrasts with the effective activity in the same assay of the relatively RARβ-specific synthetic retinoid EC19.^23^, ^53^ In contrast, GZ18 was more effective at inducing CYP26A1 (GZ25 > GZ18 > ATRA); given the role of CYP26A1 in metabolizing ATRA the promotor of this gene may be less RAR specific than the RARβ promoter.

## 3 Conclusions

The aims of this study were to combine molecular docking, based on the conformational analysis of flexible ligands, with ligand-coactivator binding and biological assays at different temporal scales to probe the relationships between structure and function of a series of synthetic retinoid compounds built around a central 2,4-disubstituted thiazole linker structure. Our major finding was that the observed ‘super’ retinoid activity of GZ25, in particular, can be understood using the combination of tools employed herein and especially when compared with less or non-active derivatives of the thiazole compounds. Thus, bearing in mind the potential uncertainties of computational predictions, the docking studies (validated by MD) predicted that GZ25 should be a better ligand for each of the RARs, except perhaps RARγ, on the basis of improved interactions of the bulkier hydrophobic region with H12 and the pocket opening. The docking work also indicated that a bulky hydrophobic region (e.g., TTN) should favor interactions with H12 and coactivator recruitment, independent of esterification of the carboxylate function. Conversely, the predicted selectivity for RARα, suggested by hydrogen bonding to Ser232 from the docking studies, was not supported by the ligand binding assays, with the possible exception of GZ18; moreover, the selectivity of GZ18 for RARβ suggested by other reports was not supported by ligand binding studies.^31^ Indeed, in biological assays, GZ25 showed strong activity, *i.e.,* perhaps super-retinoid activity being considerably better than ATRA with respect to inducing neuronal differentiation. GZ25 may, therefore, be an ideal candidate as a stable, highly active surrogate for ATRA. In contrast, the other GZ synthetic retinoids had low activity in most of the biological assays, with evidence for the induction of differentiation to non-neuronal phenotypes. The higher than expected affinity for RARβ and good activity for coactivator recruitment shown by the ester of GZ25 may make it a good starting point for designing novel RAR ligands that are genuinely RARβ-specific.

In terms of wider conclusions, it seems that while molecular docking, informed by detailed conformational analysis of potential ligands, is a powerful approach for aiding ligand design, providing good predictive value, there are some limitations. Thus, with respect to the design of RAR ligands, overall shape and interactions with H12 are important for high biological activity, and ligand affinity may be driven by polar group interactions, with interactions along the length of the ligand structure apparently of lesser importance. Uncovering the key structural and conformational effects that govern receptor binding, and hence, the impacts on downstream biology, demands a more detailed picture and analysis of the synthetic retinoid structures versus functions, since these depend on the physical separation and type of interactions that operate deep within the LBD and around the entry portal.

Further work to apply this approach and develop increasingly selective ranges of RAR-binding ligands is underway and will be reported in due course.

## 4 Materials and methods

### 4.1 Retinoid solutions

Synthetic retinoids were synthesized as reported previously,^29^ and ATRA was purchased from Sigma Aldrich. All compounds were dissolved in DMSO to a concentration of 10 mM. Stock solutions were stored at −20 °C, in the dark, and defrosted in a water bath set at 37 °C prior to use.

### 4.2 Cell culture

Human pluripotent TERA2.cl.SP12 embryonal carcinoma stem cells and SH-SY5Y human neuroblastoma cells were cultured and passaged as reported previously using low lighting conditions to minimize ATRA degradation/isomerization.^8^, ^53^

### 4.3 Flow cytometry

The expression of specific cell surface protein markers (SSEA-3, TRA-1–60, A2B5) on TERA2.cl.SP12 was analyzed using flow cytometry as described previously.^53^ Briefly, TERA2.cl.SP12 cells, 2 × 10^5^ per 25 cm^2^ flask, were seeded 12–24 h before treatment with 10 μM retinoid for 7 days. The primary antibodies used were SSEA-3 (diluted 1:10, University of Iowa Hybridoma Bank), TRA-1–60 (diluted 1:50, Abcam) and neural cell marker A2B5 (diluted 1:40, R&D Systems). Labeled cells were analyzed in a Guave EasyCyte Plus System (Millipore) flow cytometer and thresholds determining the numbers of positively expressing cells were set against the negative control antibody, P3X.

### 4.4 Gene expression analysis

Real time PCR was carried out immediately after treatment on both cell line lysates with 0.25% trypsin–EDTA. TERA-2.cl.SP12 cells were seeded at a density of 1 × 10^6^ cells per 25 cm^2^ flask, 12–24 h before treatment. Cells were treated with each retinoid at a concentration of 10 μM for 3, 5 and 7 days. SH-SY5Y cells were seeded at a density of 1 × 10^6^ cells per 25 cm^2^ flask, 12–24 h before treatment. Cells were treated with each retinoid at a range of concentrations (1, 0.1, 0.01 and 0.001 μM) and at different time scales (2–12 h). Commercial RNA extraction kits (Qiagen) and reverse transcription (Applied Biosystems) kits were purchased and procedures used according to the manufacturer instructions. Real time PCR was performed using the TaqMan® Universal PCR Master Mix (Life technologies) and TaqMan® gene expression system (Applied Biosystems) based on probe sets to the specific genes to be analysed. Genecodes used are as follows: RARβ (Hs00233407_m1), CYP26-A1 (Hs00175627_m1), RARα (Hs00940448_g1), RARγ (Hs01559234_m1), PAX-6 (Hs01088112_m1), NeuroD1 (Hs01922995_s1). GADPH (Hs02758991_g1) and ACTB (Hs99999903_m1) were used as internal control genes for TERA-2.cl.SP12 and SH-SY5Y, respectively.

### 4.5 Immunocytochemistry

TERA2.cl.SP12 cells were seeded at 5000 cells per well on poly-d-lysine (25 μg/ml) coated cover slips in 6-well plates. Cells were treated with each retinoid at a concentration of 10 μM for 7, 14 and 21 days (the media was changed every 3–4 days). Cells were also treated with 1% DMSO for the same time periods as a positive control. At the end of the treatment period, cells were fixed (4% para-formaldehyde in PBS for 30 min at room temperature (RT) and rinsed with PBS. One set of experiments was used for the cell surface marker A2B5 (diluted 1:100, R&D systems), and the other set was prepared for intracellular staining using 1% Triton-X-100 (Sigma Aldrich) in PBS as a permeabilizing agent for 10 min at RT. Non-specific labeling was blocked by incubation with a solution of 1% goat serum (Sigma Aldrich) containing 0.2% Tween-20 (Sigma Aldrich) in PBS for one hour at RT. The primary antibodies β-III tubulin antibody (TUJ-1) (1:200, Affymetrix eBioscience) and CK-8 antibody (1:500, Affymetrix eBioscience) were diluted in blocking solution and incubated with the cells for 1 h at RT. After washing three times for 15 min with PBS, cells were incubated for 1 h in the dark with anti-mouse FITC-conjugated secondary antibody IgM (1:128, Sigma Aldrich) for A2B5 staining, and anti-mouse Alexafluor 488 IgG (1:600, Invitrogen) for TUJ-1 and CK-8 staining. Hoechst 33,342 nuclear staining dye (1:1000, Molecular Probes) was dissolved in blocking solution after the secondary antibody staining step for nuclear staining.

### 4.6 Imaging

Immunocytofluorescent images of fixed cells were visualized using a Leica SP5 confocal microscope. Scale bars are shown at 25 μm.

### 4.7 Receptor binding assays

The binding interaction of each retinoid with RARα, β and γ were determined *in vitro* by time-resolved fluorescence resonance energy transfer (TR-FRET) using the LanthaScreen TR-FRET RARα, RARβ, and RARγ co-activator assays according to the manufacturer’s (Invitrogen™) instructions.^23^ This uses a terbium-labeled anti-GST antibody, a fluorescein-labeled co activator peptide, and RARα/β/γ ligand-binding domains (LBD) that are tagged with glutathione-S-transferase (GST) in a homogenous assay format. All experiments were performed in black, 384-well low-volume plates in the dark at RT with a 4 h incubation time. The final assay volume was 20 μL and all dilutions were carried out using TR-FRET assay buffer, with a final DMSO concentration of 1%. A mixture of either 3.5 nM GST-RAR-α-LBD or 2.5 nM GST-RAR-β-LBD or 3 nM GST-RAR-γ-LBD with 62.5 nM Tb-anti GST antibody and 30 μM fluorescein-labeled peptide and retinoid or DMSO control was added to each of the wells. Each ligand assay was performed in duplicate and measured using a PHERAstar FS Microplate Reader (BMG Labtech, Ortenberg, Germany) with instrument settings as described in the manufacturer’s instructions for LanthaScreen assays. The TR-FRET signal was expressed as the ratio of the signals at 520 nm and 490 nm. The data were fitted to a three-parameter ligand-binding curve using SigmaPlot (version 12.5, Systat Software Inc., San Jose CA) and normalized to the lower asymptote of each binding curve. The TR-FRET binding assay produces symmetrical sigmoid curves varying in location of the mid-point (EC_50_) and upper asymptote. Since the assay involves interactions between ligand and LBD, and ligand-dependent interactions between the LBD and fluorescein-labeled co activator, the binding assay data were interpreted by simulating these coupled chemical reactions using the biochemical simulator COPASI version 4.3.^23^, ^54^

### 4.8 Molecular modeling

Molecular structures of all compounds (ATRA, GZ18, GZ22, GZ23, GZ24 and GZ25) were generated using Spartan ‘14 (Wavefunction Inc., Irvine, CA).^37^ These were minimized using a molecular mechanics force field, followed by semi-empirical molecular orbital (AM1) methods to generate a conformer distribution *in vacuo*. The generated conformations of each compound were then checked by re-minimization using a higher level of theory (Hartree-Fock, 3-21G) to ensure that the generated conformer distribution was realistic. All possible conformations of all compounds were saved as *.mol2* files for use in the docking studies.

### 4.9 Docking

Docking calculations were conducted according to a previously reported procedure,^23^ in which all of the output conformations from the molecular modeling studies were docked into RARα, β and γ, in order to more-completely sample the available conformational space of the ligand.^55^ Docking calculations were conducted using GOLD.^39^

Crystal structures were retrieved from the RCSB protein data bank as PDB files (3KMR^20^ for RARα, 1XAP^30^ for RARβ and 2LBD^10^ for RARγ). The bound ligands were removed from the structures, and used only as a positional reference. Hydrogen atoms were added to protein residues using the default GOLD settings.^39^ All solvent molecules were removed. Active site residues were selected within a diameter of 15 Å, measured from a selected point at the center of the ligand position, and therefore included the entire binding pocket. It is important to note that the carboxylic acid moiety was considered as a carboxylate, rather than protonated, as this is more realistic with respect to physiological pH, and to the likelihood that the negative charge is stabilized by the closely positioned polar residues in the binding pocket.^56^, ^57^ ChemScore was chosen as the most appropriate target function in the genetic algorithm to balance between computational speed and the reliability of the GOLD predictions for the possible conformations of the different retinoids binding to RARs.^58^ The genetic algorithm parameters were based on previous examples of docking hydrophobic ligands: population size 100; number of islands 5; niche size 2; selection pressure 1.1; migrate 2; and number of operators 100,000.^59^ A search efficiency of 200% was used, which dictates maximum ligand flexibility. During the docking process no limit was placed on the number of binding poses retained, though typically 3–10 solutions were retained by the genetic algorithm.

### 4.10 Molecular dynamics simulation

The docked structures of each ligand with the three isoforms of RAR were used as starting models. Any remaining solvent molecules were removed. Missing loops were built with MODELLER (v9.19)^60^, ^61^ via UCSF Chimera.^62^ The final protein structures covered residues 182–415 for RARα,^20^ 182–411 for RARβ,^30^ and 188–419 for RARγ.^10^

The ligands were parameterised using Gasteiger point charges in the AMBER ff99sb^63^ forcefield for simulation using Antechamber,^64^ and prepared for GROMACS using ACPYPE.^65^ The complexes for each isoform-ligand pair were then prepared in GROMACS,^42^ parameterised in ff99sb and neutralised with sodium and chloride ions to a final concentration of 0.1 M, prior to being subject to minimisation and equilibration. Briefly, a steepest descent gradient was used to minimise the complexes, which all converged on an Fmax <1000 kJ mol^−1^ nm^−1^ in less than 1200 steps. The complexes were then subject to 200 ps equilibration in the NVT ensemble using a 2 fs timestep and Nose-Hoover temperature coupling at 300 K; all atoms were subject to position restraints. Subsequent equilibration in the NPT ensemble was also conducted under position restraints at a 2 fs timestep, with Parinello-Rahman barostat at 1 bar. Finally, position restraints were released and two parallel production MD simulations were conducted for a 10 ns time period. The *g_mmpbsa*^43^ package was used to calculate binding energies between ligand and protein, and to quantify per-residue contributions to binding. The final 2 ns of converged simulation time was used for each of the simulations, totalling 201 snapshots from 8 to 10 ns.

## Author contributions

HH carried out initial docking studies and all laboratory work, DRC developed and applied the statistical docking analyses, NJT conducted molecular dynamics simulations, AW, CR, EP and SP designed different aspects of the study, all authors wrote the paper and contributed important intellectual content.

## A Supplementary data

Supplementary data Biochemical model of the TR-FRET binding assay; calculated conformer distributions of GZ18, GZ22, GZ23, GZ24 and GZ25; clustering analysis of thiazole ring orientation; docking/MD comparison, X-ray crystallography; additional references.
