# Supplementary material for: Probing biological activity through structural modelling of ligand-receptor interactions of 2,4-disubstituted thiazole retinoids
Source: Bioorg Med Chem. 2018 May 1;26(8):1560–72. doi: 10.1016/j.bmc.2018.02.002 (PMC5933457; doi:10.1016/j.bmc.2018.02.002)
Supplement: Supplementary data — Biochemical model of the TR-FRET binding assay; calculated conformer distributions of GZ18, GZ22, GZ23, GZ24 and GZ25; clustering analysis of thiazole ring orientation; docking/MD comparison, X-ray crystallography; additional references. [file mmc1.docx]

**SUPPLEMENTARY INFORMATION**

**Probing biological activity through structural modelling of ligand-receptor interactions of 2,4-disubstituted thiazole retinoids**

Hesham Haffez,^a^ David R. Chisholm,^b^ Natalie J. Tatum,^c^ Roy Valentine,^d^ Christopher Redfern,^c*^ Ehmke Pohl,^b,e^ Andrew Whiting^b*^ and Stefan Przyborski^e^

*^a^Department of Biochemistry and Molecular Biology, Pharmacy College, Helwan University, Cairo, Egypt.*

^b^Department of Chemistry, Centre for Sustainable Chemical Processes, Durham University, South Road, Durham, DH1 3LE, UK.

*^c^Northern Institute for Cancer Research, Medical School, Newcastle University, Newcastle upon Tyne, NE2 4HH, UK.*

*^d^High Force Research Limited, Bowburn North Industrial Estate, Bowburn, Durham, DH6 5PF, UK*

^e^Department of Biosciences, Durham University, South Road, Durham DH1 3LE, UK.

Table of Contents

[1. Biochemical model of the TR-FRET binding assay 2](#_Toc504884698)

[2. Calculated conformer distributions of GZ18, GZ22, GZ23, GZ24 and GZ25 3](#_Toc504884699)

[3. Clustering analysis of thiazole ring orientation 10](#_Toc504884700)

[4. Docking *versus* molecular dynamics comparisons 11](#_Toc504884701)

[5. X-ray crystallography 12](#_Toc504884702)

[6. References 14](#_Toc504884703)

## Biochemical model of the TR-FRET binding assay

The readout of the Lanthascreen TR-FRET binding assay is based on a Forster resonance energy transfer (FRET) signal generated from the interaction between a terbium-labelled anti-glutathione-S-transferase (GST) antibody and a fluorescein-labelled coactivator peptide. The RAR ligand-binding domain (LBD) is a GST fusion protein and interaction between the LBD and the fluorescein-labelled coactivator peptide is driven by the binding of ligand to the LBD, and detected by the FRET signal from the terbium/fluorescein interaction when the terbium-labelled anti-GST binds the LBD fusion protein. The output of the assay thus relies on two binding interactions: ligand with LBD, and ligand-bound LBD with the coactivator peptide. As both interactions may be affected by the fit of ligand to the binding pocket of the LBD, we used the biochemical pathway simulator COPASI^1^ to build a simple model of how the FRET signal output in the assay will vary according to differential changes in the kinetics of the two binding interactions. Reactions were modelled deterministically using mass-action kinetics described by ordinary differential equations (ODE) within COPASI.^1^ This model enables us to assess the effect of different rate constants and varying ligand concentration on the formation of the final ternary complex that is the read-out from the Lanthascreen TR-FRET assay. The two coupled reactions are controlled by the four rate constants k_1_-k_4_. Rate constants from analogous ligand-nuclear receptor systems^2,3,4^ were used as the starting point for calculating the EC_50_ (half maximal effective concentration) for ATRA. These rate constants were then altered individually or together to model different possible scenarios.^5^ The simulation results show that the k_1_/k_2_ ratio, reflecting the affinity of ligand for LBD, directly affects EC_50_ values, while alteration in k_3_/k_4_, reflecting the affinity of co-activator for the ligand-LBD complex (LR), changes both the EC_50_ and upper asymptote.

## Calculated conformer distributions of GZ18, GZ22, GZ23, GZ24 and GZ25

**Table 1:** Conformer distribution of GZ18 in order of lowest energy. Distribution was calculated using the AM1 forcefield, and energies are shown from this calculation. The results were further validated at a higher level of theory (HF, STO-3G), and no obvious differences were exhibited. Energies are expressed in kJ/mol, and were calculated using Spartan 14. Each of these calculated conformations was used as a starting point for the docking process.

| 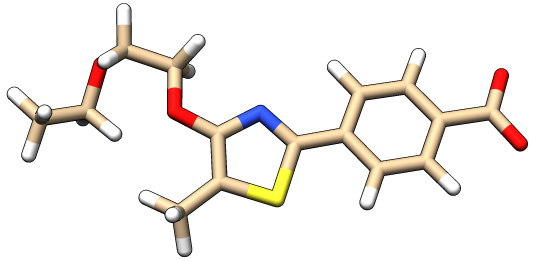  Conformation 1: -605.98 (lowest) | | 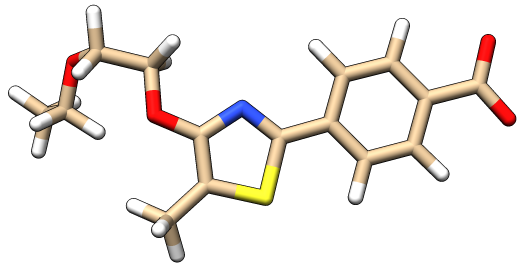  Conformation 2: -604.40 |
| --- | --- | --- |
| 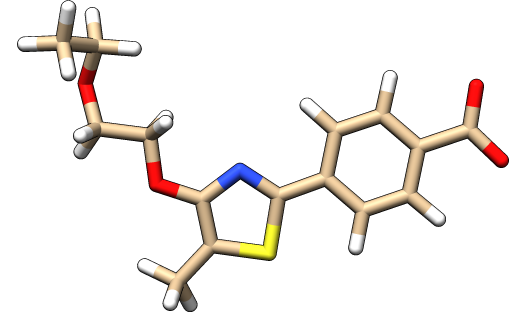  Conformation 3: -601.24 | 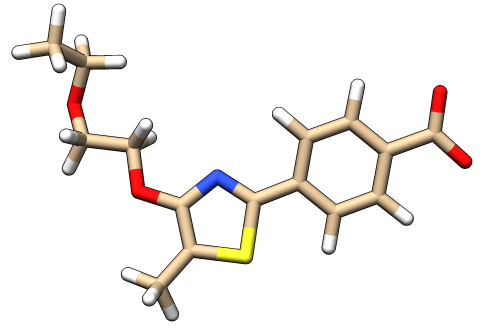  Conformation 4: -601.20 |  |
| 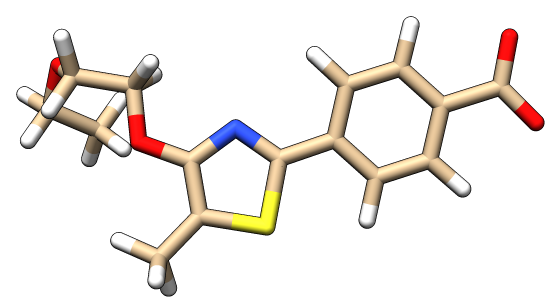  Conformation 5: -601.16 | 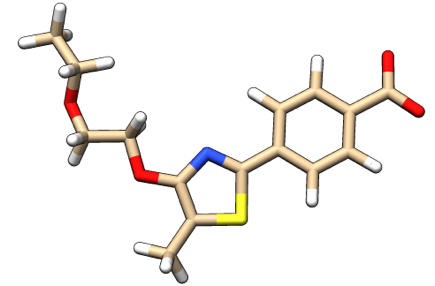  Conformation 6: -600.11 |  |
| 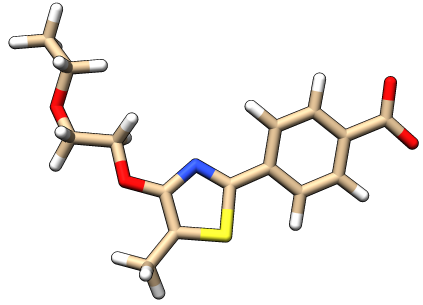  Conformation 7: -599.95 | 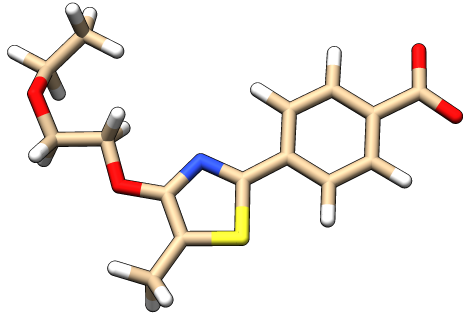  Conformation 8: -599.06 |  |
| 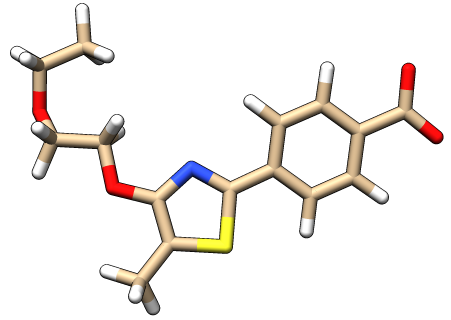  Conformation 9: -598.46 | 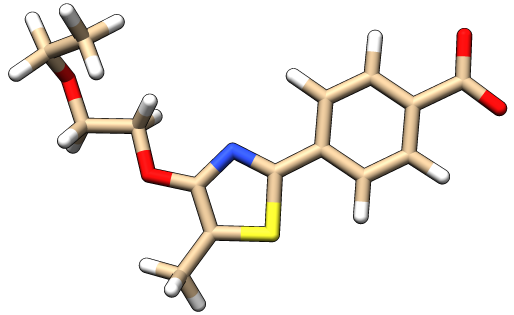  Conformation 10: -598.18 |  |
| 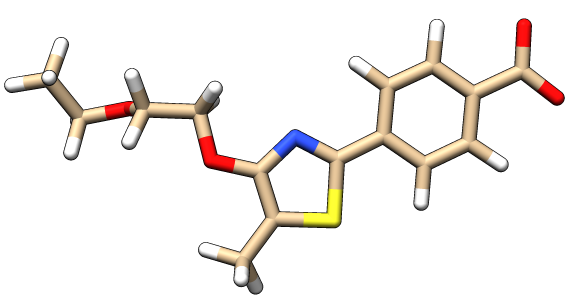  Conformation 11: -598.18 | 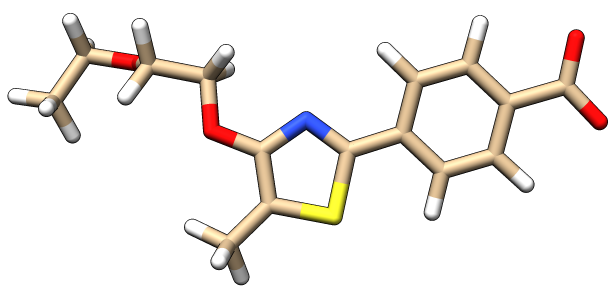  Conformation 12: -596.44 |  |
| 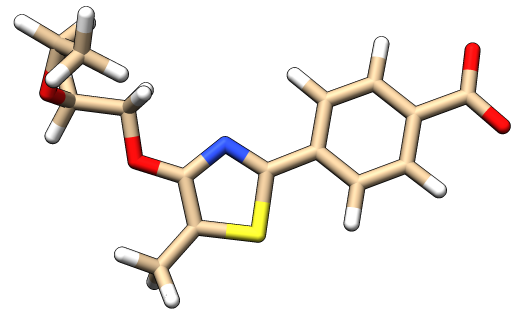  Conformation 13: -596.27 | 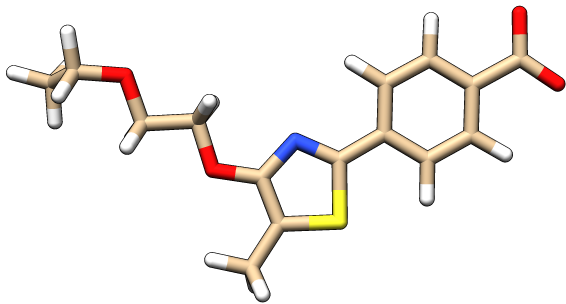  Conformation 14: -595.59 |  |
| 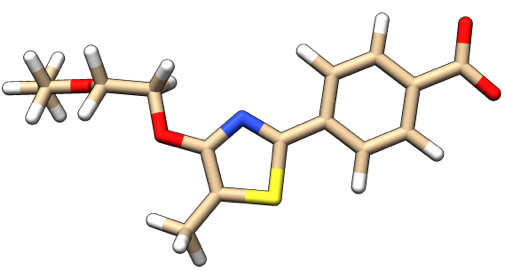  Conformation 15: -595.06 | 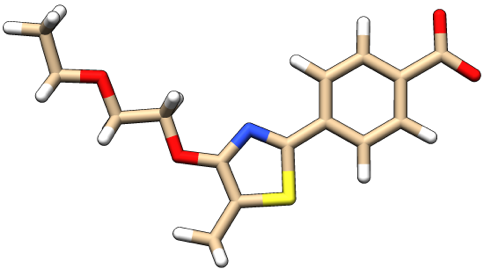  Conformation 16: -594.06 |  |

**Table 2:** Conformer distribution of GZ22 in order of lowest energy. Distribution was calculated using the AM1 forcefield, and energies are shown from this calculation. The results were further validated at a higher level of theory (HF, STO-3G), and no obvious differences were exhibited. Energies are expressed in kJ/mol, and were calculated using Spartan 14. Each of these calculated conformations was used as a starting point for the docking process.

| 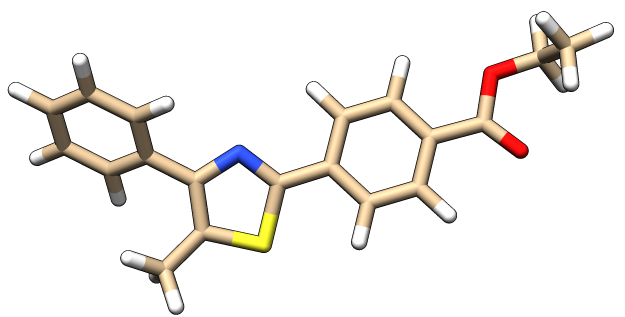  Conformation 1: -18.55 (lowest) | | 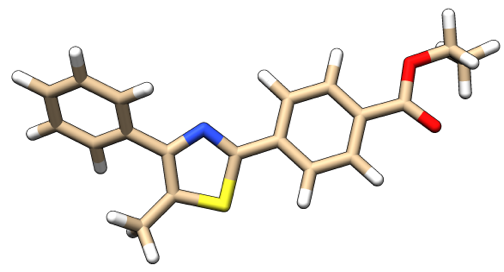  Conformation 2: -18.55 |
| --- | --- | --- |
| 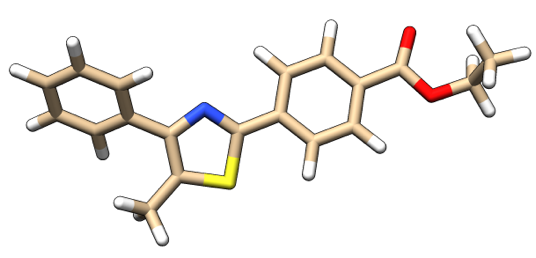  Conformation 3: -18.30 | 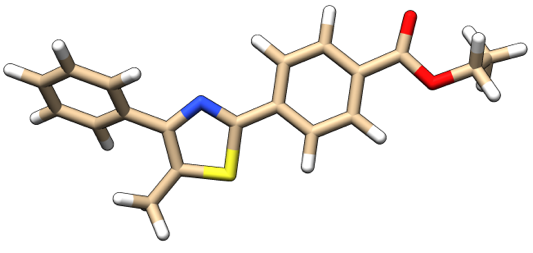  Conformation 4: -18.30 |  |
| 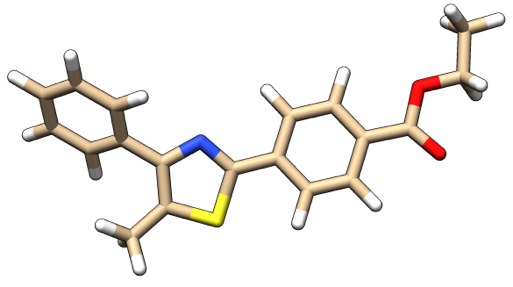  Conformation 5: -18.26 | 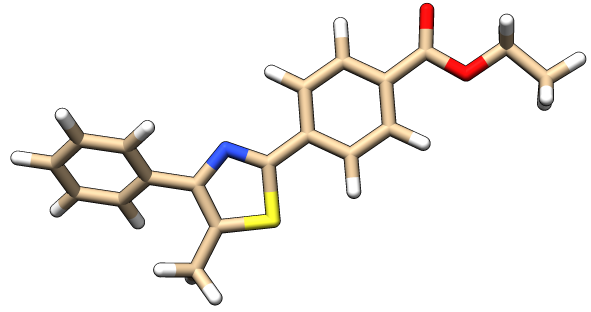  Conformation 6: -17.95 |  |

**Table 3:** Conformer distribution of GZ23 in order of lowest energy. Distribution was calculated using the AM1 forcefield, and energies are shown from this calculation. The results were further validated at a higher level of theory (HF, STO-3G), and no obvious differences were exhibited. Energies are expressed in kJ/mol, and were calculated using Spartan 14. This calculated conformation was used as a starting point for the docking process.

| 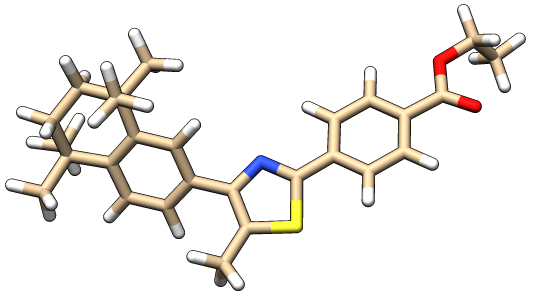  Conformation 1: -149.34 (lowest) | | 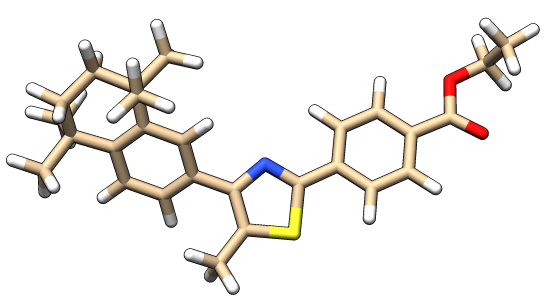  Conformation 2: -149.34 |
| --- | --- | --- |
| 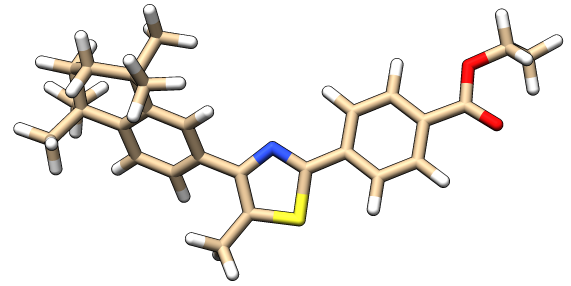  Conformation 3: -149.33 | 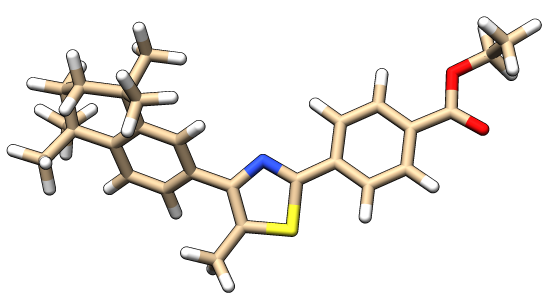  Conformation 4: -149.31 |  |
| 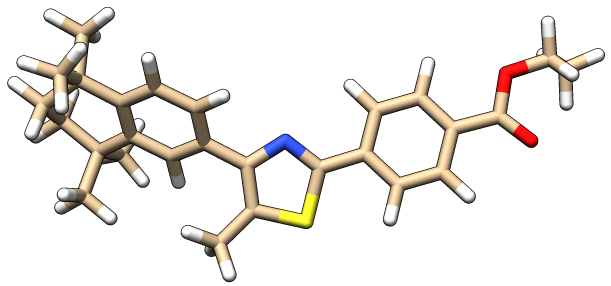  Conformation 5: -149.22 | 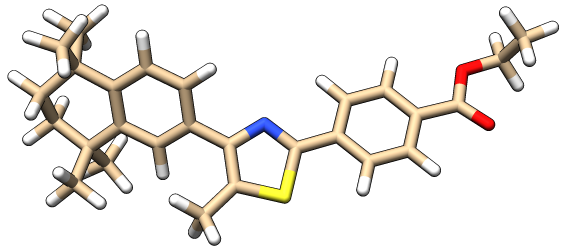  Conformation 6: -149.21 |  |
| 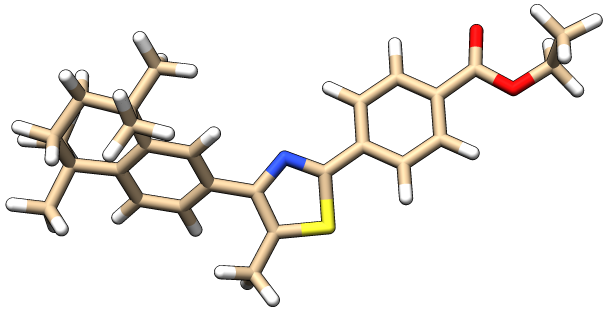  Conformation 7: -149.12 | 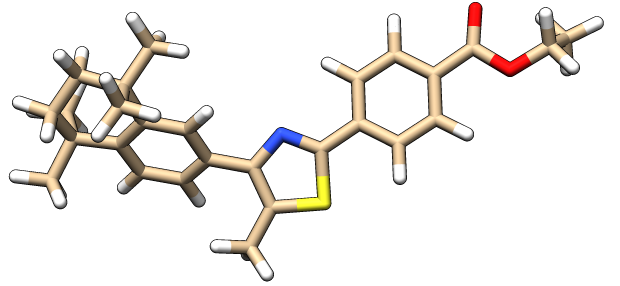  Conformation 8: -149.11 |  |
| 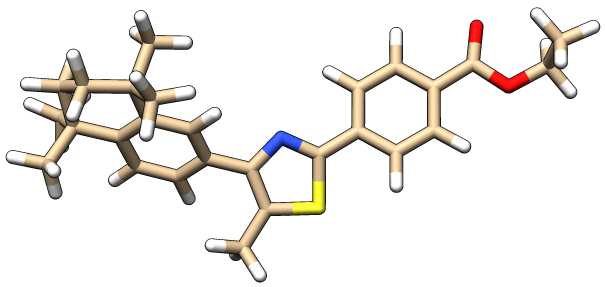  Conformation 9: -149.09 | 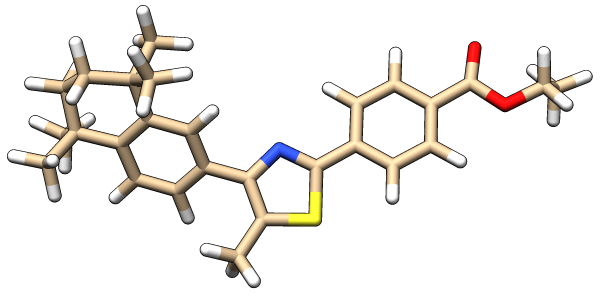  Conformation 10: -149.09 |  |
| 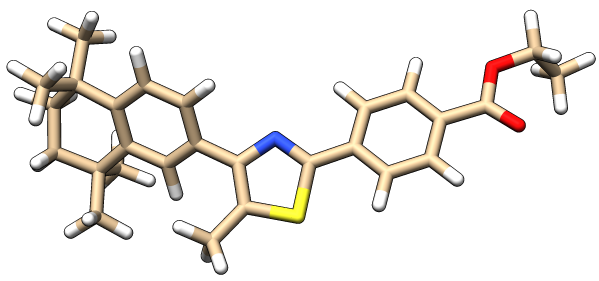  Conformation 11: - 149.05 | 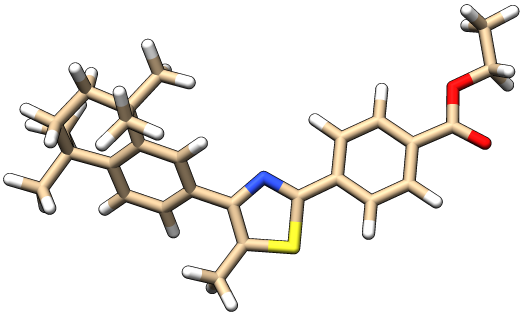  Conformation 12: -149.04 |  |
| 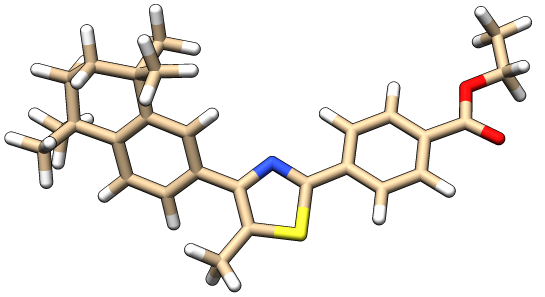  Conformation 13: -149.04 | 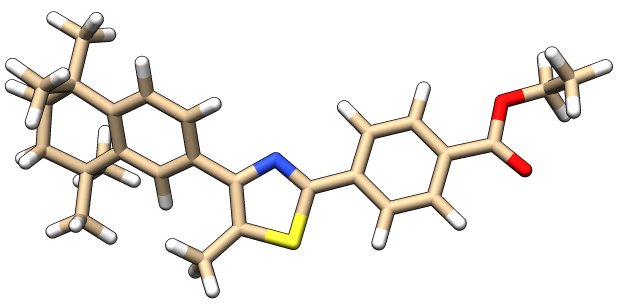  Conformation 14: -149.00 |  |
| 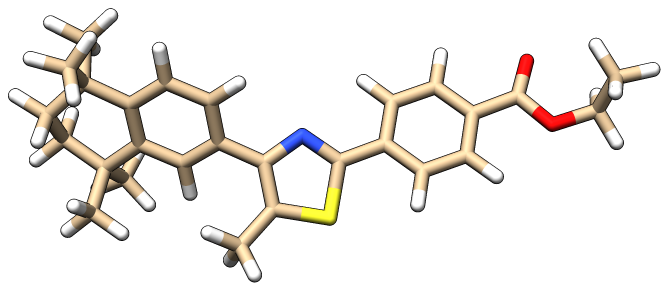  Conformation 15: - 148.98 | 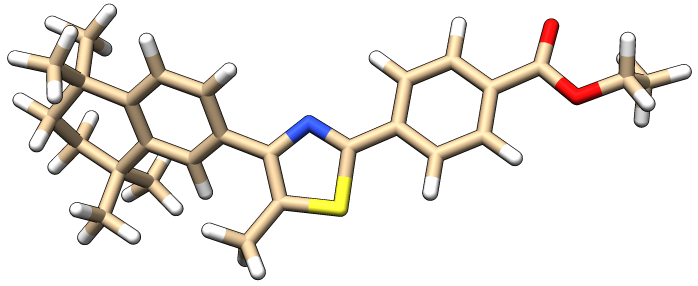  Conformation 16: -148.95 |  |
| 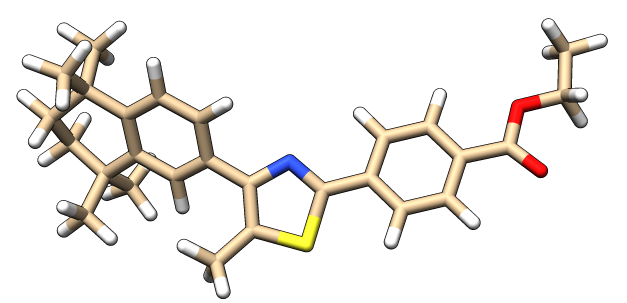  Conformation 17: -148.94 | 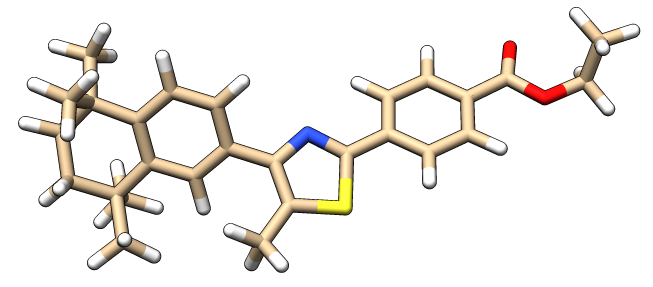  Conformation 18: -148.81 |  |
| 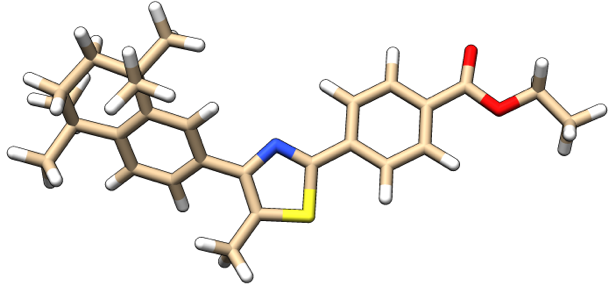  Conformation 19: -148.79 | 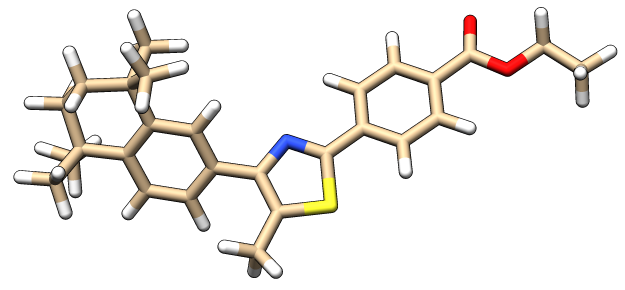  Conformation 20: -148.78 |  |
| 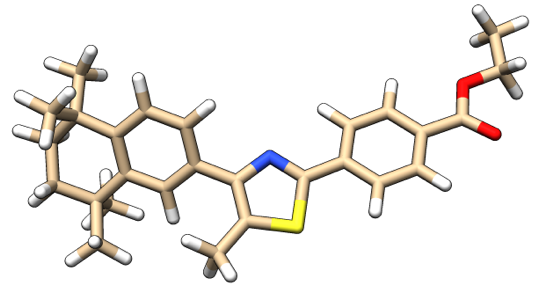  Conformation 21: -148.77 | 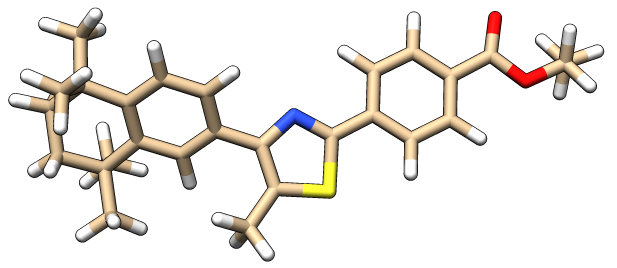  Conformation 22: -148.75 |  |
| 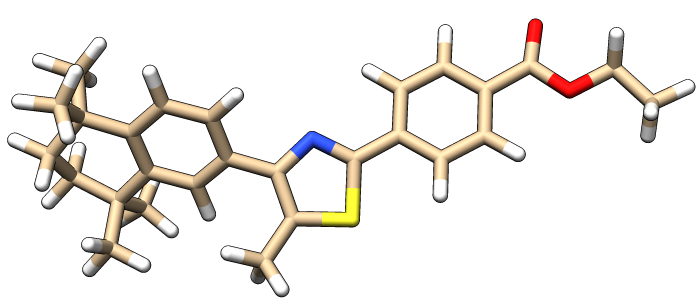  Conformation 23: -148.66 | 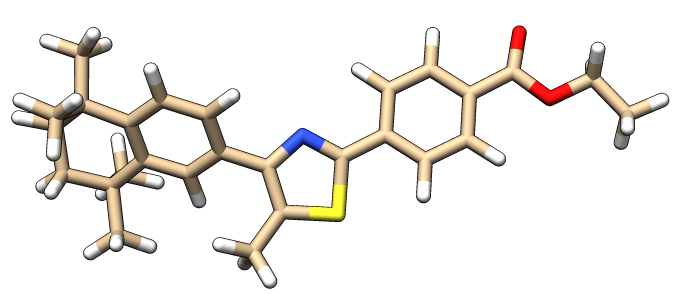  Conformation 24: -148.49 |  |

**Table 4:** Conformer distribution of GZ24 in order of lowest energy. Distribution was calculated using the AM1 forcefield, and energies are shown from this calculation. The results were further validated at a higher level of theory (HF, STO-3G), and no obvious differences were exhibited. Energies are expressed in kJ/mol, and were calculated using Spartan 14. This calculated conformation was used as a starting point for the docking process.

| 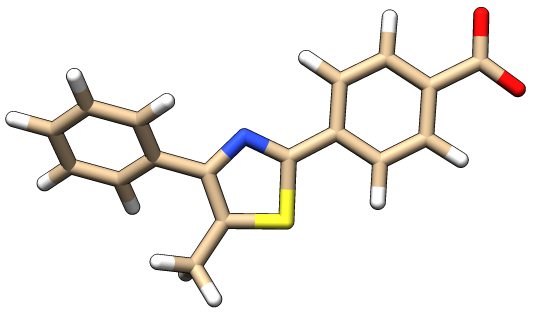  Conformation 1: -119.05 (lowest) |
| --- |

**Table 5:** Conformer distribution of GZ25 in order of lowest energy. Distribution was calculated using the AM1 forcefield, and energies are shown from this calculation. The results were further validated at a higher level of theory (HF, STO-3G), and no obvious differences were exhibited. Energies are expressed in kJ/mol, and were calculated using Spartan 14. Each of these calculated conformations was used as a starting point for the docking process.

| 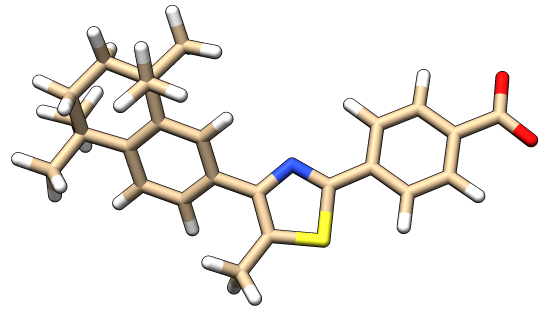  Conformation 1: -249.03 (lowest) | | 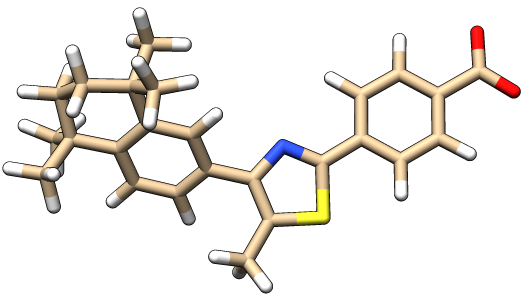  Conformation 2: -249.01 |
| --- | --- | --- |
| 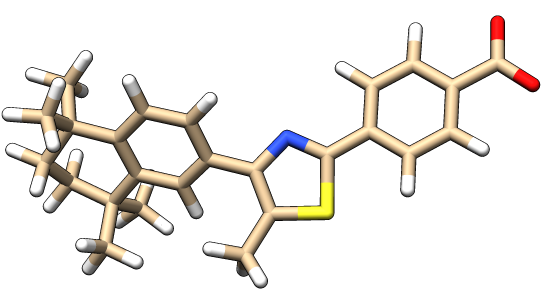  Conformation 3: -248.61 | 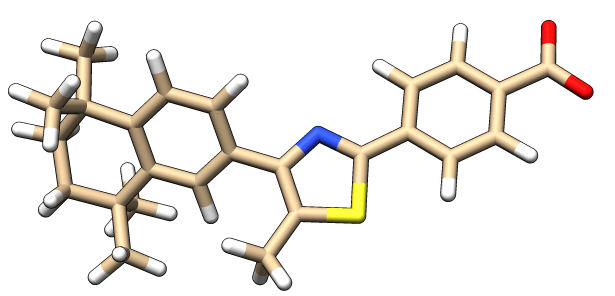  Conformation 4: -248.43 |  |

## Clustering analysis of thiazole ring orientation

Tables 6-8 show a clustering analysis of the preferred orientations of the thiazole ring of GZ18, GZ22, GZ23, GZ24 and GZ25 with respect to the RARα/β/γ binding pocket according to the docking calculations.

**Table 6:** Clustering analysis of the orientation of the thiazole ring of GZ18, GZ22, GZ23, GZ24 and GZ25 with respect to the RARα binding pocket.

| Retinoid | Thiazole nitrogen up | Thiazole nitrogen down | Number of docking solutions |
| --- | --- | --- | --- |
| GZ18 | 100% | 0% | 50 |
| GZ22 | 100% | 0% | 18 |
| GZ23 | 100% | 0% | 90 |
| GZ24 | 100% | 0% | 3 |
| GZ25 | 100% | 0% | 13 |

**Table 7:** Clustering analysis of the orientation of the thiazole ring of GZ18, GZ22, GZ23, GZ24 and GZ25 with respect to the RARβ binding pocket.

| Retinoid | Thiazole nitrogen up | Thiazole nitrogen down | Number of docking solutions |
| --- | --- | --- | --- |
| GZ18 | 13% | 87% | 64 |
| GZ22 | 40% | 60% | 42 |
| GZ23 | 97% | 3% | 75 |
| GZ24 | 100% | 0% | 3 |
| GZ25 | 92% | 8% | 13 |

**Table 8:** Clustering analysis of the orientation of the thiazole ring of GZ18, GZ22, GZ23, GZ24 and GZ25 with respect to the RARγ binding pocket.

| Retinoid | Thiazole nitrogen up | Thiazole nitrogen down | Number of docking solutions |
| --- | --- | --- | --- |
| GZ18 | 88% | 12% | 57 |
| GZ22 | 100% | 0% | 18 |
| GZ23 | 100% | 0% | 75 |
| GZ24 | 100% | 0% | 3 |
| GZ25 | 100% | 0% | 12 |

## Docking *versus* molecular dynamics comparisons


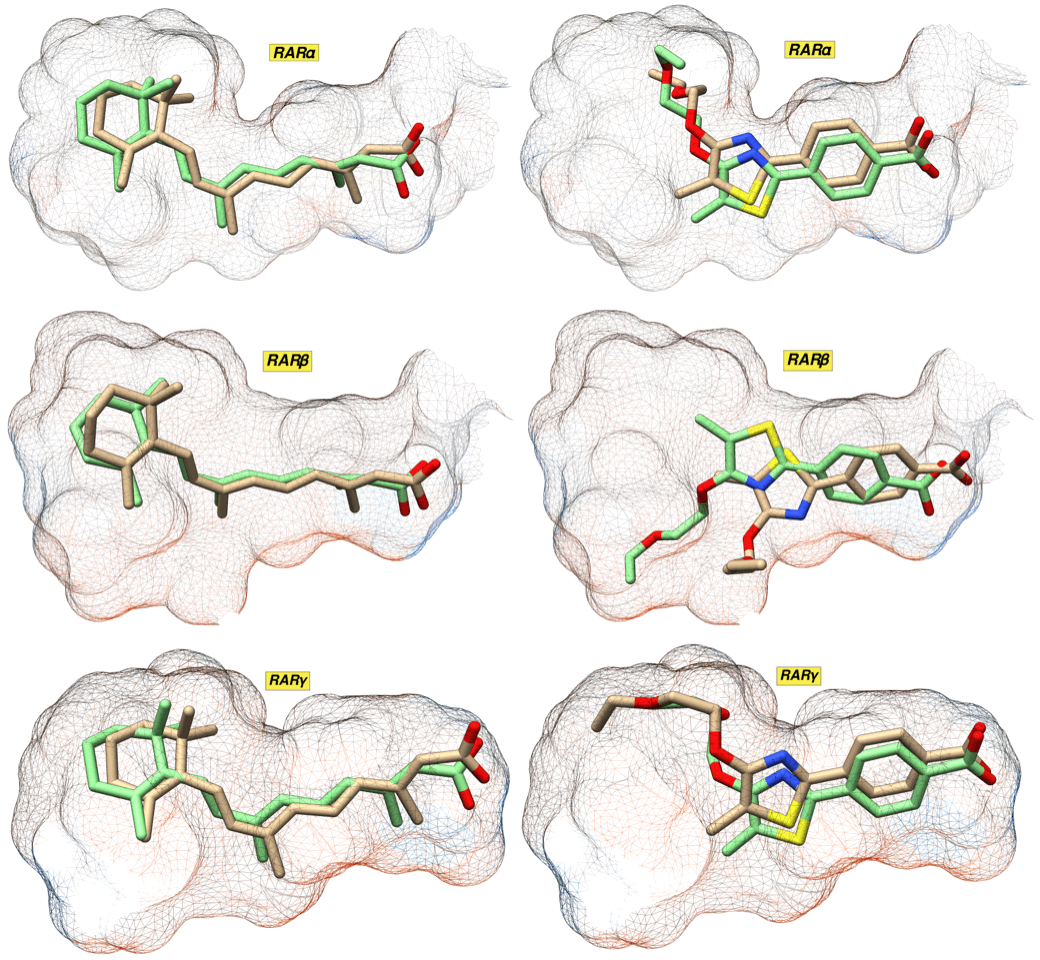


Figure 1: Comparison between the docking binding poses (green) and the MD binding poses (tan) for ATRA (left) and GZ18 (right) in each of the RARs.


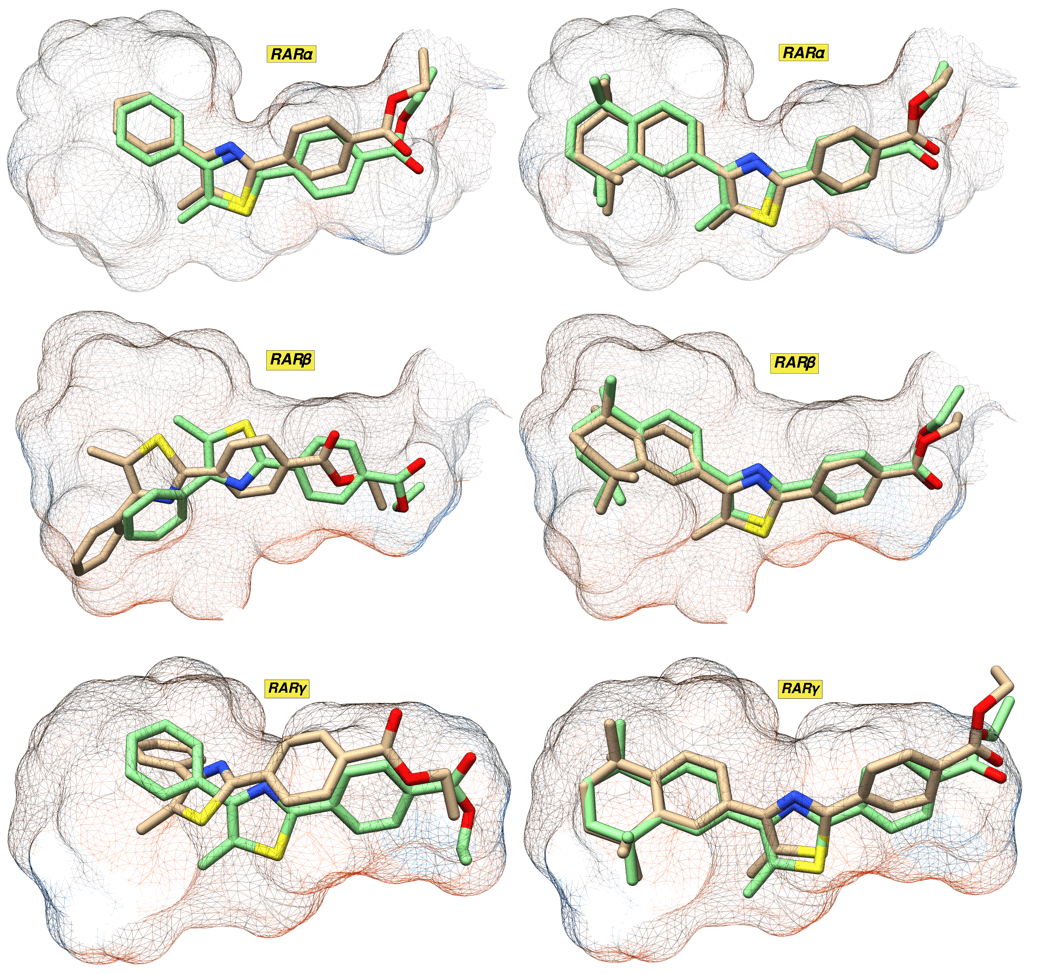


Figure 2: Comparison between the docking binding poses (green) and the MD binding poses (tan) for GZ22 (left) and GZ23 (right) in each of the RARs.

## X-ray crystallography

Single-crystal diffraction experiments were conducted on Bruker APEX-II CCD (**GZ18** and **GZ23**) and Bruker MicroStar (**GZ22**) diffractometers. Crystals were cooled using Cryostream (Oxford Cryosystems) open-flow N_2_ cryostats. The structures were solved within Olex2 by direct methods and refined by full-matrix least squares against F^2^ of all data, using SHELXTL software.^6–8^ All non-hydrogen atoms were refined anisotropically. Hydrogen atom positions were calculated geometrically and refined using the riding model. CCDC (1562378-1562380) contains the supplementary crystallographic data for this paper. The data can be obtained free of charge from The Cambridge Crystallographic Data Centre via [www.ccdc.cam.ac.uk/getstructures](http://www.ccdc.cam.ac.uk/getstructures).

| **Compound** | **GZ18** | **GZ22** | **GZ23** |
| --- | --- | --- | --- |
| Empirical formula | C_15_H_17_NO_4_S | C_19_H_17_NO_2_S | C_27_H_31_NO_2_S |
| Formula weight | 307.35 | 323.39 | 433.59 |
| Temperature/K | 120 | 100 | 120 |
| Crystal system | triclinic | monoclinic | monoclinic |
| Space group | P-1 | P2_1_/c | P2_1_/n |
| a/Å | 7.6107(4) | 9.8060(7) | 17.7889(15) |
| b/Å | 8.4285(5) | 29.958(2) | 6.0157(6) |
| c/Å | 22.8824(13) | 10.7715(8) | 22.128(2) |
| α/° | 97.519(2) | 90 | 90 |
| β/° | 96.984(2) | 94.554(2) | 97.650(3) |
| γ/° | 94.088(2) | 90 | 90 |
| Volume/Å^3^ | 1438.73(14) | 3154.4(4) | 2346.9(4) |
| Z | 4 | 8 | 4 |
| ρ_calc_g/cm^3^ | 1.419 | 1.362 | 1.227 |
| μ/mm^‑1^ | 0.24 | 1.895 | 0.161 |
| F(000) | 648 | 1360 | 928 |
| Crystal size/mm^3^ | 0.449 × 0.447 × 0.158 | 0.1 × 0.1 × 0.05 | 0.707 × 0.09 × 0.078 |
| Radiation | MoKα (λ = 0.71073) | CuKα (λ = 1.54178) | MoKα (λ = 0.71073) |
| 2Θ range for data collection/° | 5.414 to 69.08 | 5.9 to 131.262 | 4.62 to 49.558 |
| Index ranges | -12 ≤ h ≤ 12, -13 ≤ k ≤ 12, -36 ≤ l ≤ 36 | -11 ≤ h ≤ 9, -35 ≤ k ≤ 34, -12 ≤ l ≤ 12 | -20 ≤ h ≤ 20, -7 ≤ k ≤ 7, -26 ≤ l ≤ 26 |
| Reflections collected | 36744 | 30958 | 25889 |
| Independent reflections | 11667 [R_int_ = 0.0328, R_sigma_ = 0.0462] | 5324 [R_int_ = 0.0261, R_sigma_ = 0.0177] | 4004 [R_int_ = 0.0768, R_sigma_ = 0.0546] |
| Data/restraints/parameters | 11667/0/385 | 5324/0/419 | 4004/0/286 |
| Goodness-of-fit on F^2^ | 1.054 | 1.088 | 1.03 |
| Final R indexes [I>=2σ (I)] | R_1_ = 0.0507, wR_2_ = 0.1257 | R_1_ = 0.0319, wR_2_ = 0.0941 | R_1_ = 0.0556, wR_2_ = 0.1186 |
| Final R indexes [all data] | R_1_ = 0.0898, wR_2_ = 0.1450 | R_1_ = 0.0330, wR_2_ = 0.0971 | R_1_ = 0.0845, wR_2_ = 0.1319 |
| Largest diff. peak/hole / e Å^-3^ | 1.12/-0.32 | 0.23/-0.28 | 0.90/-0.81 |

**Table 9:** Crystallographic data for compounds GZ18, GZ22 and GZ23. CCDC (1562378-1562380) contains the full supplementary crystallographic data for this paper. The data can be obtained free of charge from The Cambridge Crystallographic Data Centre via [www.ccdc.cam.ac.uk/getstructures](http://www.ccdc.cam.ac.uk/getstructures).

Figure 3: X-ray molecular structure of GZ18 (one of two molecules in the asymmetric unit). Thermal ellipsoids are drawn at the 50% probability level.

Figure 4: X-ray molecular structure of GZ22 (one of two molecules in the asymmetric unit). Thermal ellipsoids are drawn at the 50% probability level.

Figure 5: X-ray molecular structure of GZ23 (one of two molecules in the asymmetric unit). Thermal ellipsoids are drawn at the 50% probability level.

## References

1. Hoops, S.; Sahle, S.; Gauges, R.; Lee, C.; Pahle, J.; Simus, N.; Singhal, M.; Xu, L.; Mendes, P.; Kummer, U. COPASI - A COmplex PAthway SImulator. *Bioinformatics* **2006**, *22*, 3067–3074.

2. Waage, P.; Gulberg, C. M. Studies Concerning Affinity. *J. Chem. Educ.* **1986**, *63* (12), 1044.

3. Kwok, K. C.; Cheung, N. H. Measuring Binding Kinetics of Ligands with Tethered Receptors by Fluorescence Polarization and Total Internal Reflection Fluorescence. *Anal. Chem.* **2010**, *82* (9), 3819–3825.

4. Lavery, D. N.; McEwan, I. J. Functional Characterization of the Native NH2 Terminal Transactivation Domain of the Human Androgen Receptor: Binding Kinetics for Interactions with TFIIF and SRC-1a. *Biochemistry* **2008**, *47*, 3352–3359.

5. Haffez, H.; Chisholm, D. R.; Valentine, R.; Pohl, E.; Redfern, C. P. F.; Whiting, A. The Molecular Basis of the Interactions between Synthetic Retinoic Acid Analogues and the Retinoic Acid Receptors. *Med. Chem. Commun.* **2017**, *8*, 578–592.

6. Sheldrick, G. M. Crystal Structure Refinement with SHELXL. *Acta Cryst.* **2015**, *C71* (1), 3–8.

7. Sheldrick, G. M. SHELXT – Integrated Space-Group and Crystal- Structure Determination. *Acta Cryst.* **2015**, *A71* (1), 3–8.

8. Dolomanov, O. V.; Bourhis, L. J.; Gildea, R. J.; Howard, J. A. K.; Puschmann, H. OLEX2 : A Complete Structure Solution, Refinement and Analysis Program. *J. Appl. Cryst.* **2009**, *42*, 339–341.
